# Alcohol consumption and the risk of all-cause and cause-specific mortality: Linear and nonlinear Mendelian randomisation study

**DOI:** 10.1093/ije/dyae046

**Published:** 2024-02-14

**Authors:** Nigussie Assefa Kassaw, Ang Zhou, Anwar Mulugeta, Sang Hong Lee, Stephen Burgess, Elina Hyppönen

**Affiliations:** 1 Australian Centre for Precision Health, University of South Australia, Adelaide, Australia; 2 Clinical & Health Sciences, University of South Australia, Adelaide, Australia; 3 School of Public Health, Addis Ababa University, Addis Ababa, Ethiopia; 4 South Australian Health and Medical Research Institute, Adelaide, Australia; 5 Medical Research Council Biostatistics Unit, University of Cambridge, Cambridge, UK; 6 Department of Pharmacology, College of Health Sciences, Addis Ababa University, Addis Ababa, Ethiopia; 7 Allied Health & Human Performance, University of South Australia, Adelaide, Australia; 8 British Heart Foundation Cardiovascular Epidemiology Unit, University of Cambridge, Cambridge, UK

**Keywords:** Alcohol consumption, Mendelian randomisation, non-linear analysis, doubly-ranked method, mortality

## Abstract

**Background:**

Many observational studies support light to moderate alcohol intake as potentially protective against premature death. We used a genetic approach to evaluate the linear and non-linear relationships between alcohol consumption and mortality from different underlying causes.

**Methods:**

We used data from 278,093 white-British UK Biobank participants, aged 37-73 years at recruitment and with data on alcohol intake, genetic variants, and mortality. Habitual alcohol consumption was instrumented by 94 variants. Linear Mendelian randomisation (MR) analyses were conducted using five complementary approaches, and non-linear MR analyses by the doubly-ranked method.

**Results:**

There were 20,834 deaths during the follow-up (median 12·6 years). In conventional analysis, the association between alcohol consumption and mortality outcomes was “J-shaped”. In contrast, MR analyses supported a positive linear association with premature mortality, with no evidence for curvature (*P*_non-linearity_ ≥ 0·21 for all outcomes). Odds ratio [OR] for each standard unit increase in alcohol intake was 1·27 (95% CI 1·16–1·39) for all-cause mortality, 1·30 (95% CI 1·10–1·53) for cardiovascular disease, 1·20 (95% CI 1·08–1·33) for cancer, and 2·06 (95% CI 1·36–3·12) for digestive disease mortality. These results were consistent across pleiotropy-robust methods. There was no clear evidence for an association between alcohol consumption and mortality from respiratory diseases or COVID-19 (1·32, 95% CI 0·96–1·83 and 1·46, 95% CI 0·99–2·16, respectively; *P*_non-linearity_ ≥ 0·21).

**Conclusion:**

Higher levels of genetically predicted alcohol consumption had a strong linear association with increased risk of premature mortality with no evidence for any protective benefit at modest intake levels.

## Introduction

The relationship between alcohol consumption and mortality is complex.^1^ Excessive alcohol intake is associated with a higher risk of premature death.^2^ However, previous observational studies suggest a “J-shaped” relationship between alcohol consumption and mortality, where light to moderate consumption has the lowest mortality, and both high levels and abstaining from alcohol are associated with an increased risk of death and morbidity.^3–6^ This has led to suggestions that light to moderate alcohol consumption might offer protection against premature death and to an ongoing debate about safe levels of alcohol intake.

Natural experiments using alcohol control policies, such as tax increases and availability restrictions, support a protective effect of lower alcohol intake on all-cause mortality^7^. However, determining the causal link between moderate habitual alcohol consumption and mortality risk is challenging and while such studies have been attempted,^8^ we are not aware of any published randomized controlled trials (RCTs). Mendelian randomisation (MR) is a statistical technique that uses naturally occurring genetic variation, typically single nucleotide polymorphisms (SNPs), to infer a causal relationship between an exposure (in this case, alcohol consumption) and an outcome (in this case, mortality risk)^9^ (Supplementary Figure S1). This approach assumes that genetic variants are not influenced by confounders and that they are randomly allocated at conception, thereby avoiding some of the limitations with traditional observational studies, such as confounding and reverse causality,^10^ and providing more robust evidence of causal links. There are several MR studies investigating the effect of alcohol intake on various health outcomes including cardiovascular diseases (CVD),^11, 12^ stroke,^13^ and Alzheimer’s disease.^14^ These studies have typically relied on linear MR approaches, and as such could not have ascertained if a modest alcohol intake indeed had a beneficial protective effect. An interesting study is that conducted by Millwood et al,^15^ which approximated average alcohol intakes based on known differences in consumption patterns between regions, with area stratified MR analyses and cross-centre comparisons suggesting similar increases in cardiovascular risk regardless of estimated alcohol intake. There is also a recent study looking at the association between alcohol intake and CVD outcomes which used a non-linear MR approach in addition to conducting standard linear analyses. This study suggested a quadratic pattern to be the best fit for the associations between alcohol consumption and hypertension, coronary heart disease, and all-cause mortality.^16^ However, their analyses did not address associations of alcohol across cause-specific mortalities, and the inference on the shape of the association remains uncertain as subsequent methodological work suggests there has been likely violations to model assumptions specific to the statistical approach used.^17^

In this study, we examine the genetic evidence for a causal relationship between alcohol consumption and mortality risk, including all-cause mortality and deaths caused by CVD, cancer, respiratory disease, digestive disease, and COVID-19. Our study used data from up to 278,093 UK Biobank participants with information on alcohol consumption, genetic variants, and mortality. The analyses were conducted using multiple complementary approaches to enhance the robustness of the findings.

## Methods

The UK Biobank is a longitudinal study that initially included data from more than 500,000 participants from the general population of the United Kingdom. Participants were recruited between March 2006 and July 2010, aged 37 to 73 years.^18^ In this study, we restricted the study population to individuals of European ancestry who identified as white British, who were unrelated,^19^ who had consistent information about self-reported and genetic sex and complete data on alcohol consumption, mortality, and covariates, leaving 278,093 participants for our analyses (Supplementary Figure S2). The UK Biobank obtained ethical approval from the National Information Governance Board for Health and Social Care and North West Multicentre Research Ethics Committee (11/NW/0382). This study was conducted under the application number 20175. All participants gave informed consent and those who withdrew were excluded from the analysis.

We obtained mortality data from National Health Service (NHS) Digital and the NHS Central Register (https://www.ukbiobank.ac.uk/) until November 12, 2021. Our study outcomes included both all-cause mortality and cause-specific mortalities from CVD, cancer, digestive, respiratory, and COVID-19 diseases. We defined causes of death using the tenth revision of the International Classification of Diseases (ICD)^20^ (Supplementary Table S1). As a sensitivity analysis to reduce potential bias from competing causes, we also restricted our analyses of all-cause mortality to deaths prior to the COVID-19 pandemic, including deaths until January 1, 2020.

Alcohol consumption was reported at baseline assessment using a touchscreen questionnaire. Participants reported their intake of different types of alcoholic drinks, and we calculated the amount of alcohol consumed as grams per day, by adding up the average consumption of various types of beverages. Intakes were reported as units of alcohol assuming the UK standard (1 unit = 8 grams of pure alcohol^21^ (Supplementary methods). Information on covariates was collected through self-reported touchscreen questionnaires, except for Townsend deprivation index (TDI) which was derived based on participants’ postcodes (Supplementary methods).

Genetic variants were identified based on a recent genome-wide association (GWAS) meta-analysis on individuals of European ancestry.^22^ From the 99 SNPs associated with alcohol consumption, we used the 94 SNPs that had a directionally consistent association with alcohol intake in the UK Biobank (Supplementary Table S2). We extracted the SNPs from the UK Biobank and calculated weighted genetic risk scores (GRS) taking the weights from the corresponding SNP-alcohol consumption association estimates in the original discovery sample (Supplementary methods).^22^

## Statistical Analysis

We used logistic regression to investigate the association of reported alcohol consumption with mortality. We used fractional polynomial models to determine the appropriate functional form comparing model-fit between the best-fitting fractional polynomial model and the linear model, using the likelihood ratio test.^23^ All models were adjusted for age, sex, education level, assessment centre, TDI, body mass index (BMI), smoking, physical activity, and self-perceived health and long-term illness.

We conducted linear and non-linear MR analyses to examine the causal relationship between alcohol consumption and mortality, with the latter used to assess evidence for curvature (Supplementary Figure S3). In both approaches, causal association was interrogated using the genetic score for alcohol intake, adjusting for age, sex, assessment centre, SNP array, birth location, and top 40 genetic principal components. In the linear MR analysis, the ratio-of-coefficients method was used to compute causal estimates,^24^ which requires GRS-exposure and GRS-outcomes association estimates as inputs. We used linear and logistic regression to obtain GRS-alcohol and GRS-mortality association estimates from the UK Biobank, respectively. In the non-linear MR analysis, we first stratified the UK Biobank sample into 25 strata using the doubly-ranked stratification method,^25^ where strata are formed by first ranking participants into pre-strata based on their level of GRS and then by ranking participants within each pre-stratum according to their level of alcohol intake. Within each stratum, we computed stratum-specific GRS-alcohol and GRS-outcomes effect estimates, and then applied the ratio-of-coefficients method (as described in the linear MR analysis) to calculate the localized average causal effect (LACE) estimate. To assemble the alcohol-mortality curve, we then carried out meta-regression of LACE estimates against the stratum-specific mean alcohol consumption using a fractional polynomial function. The best-fitting fractional polynomial model was determined by the likelihood ratio test. The fractional polynomial test was reported for non-linearity, which compares the best-fitting fractional polynomial of degree 1 against the linear model.^25^ Furthermore, for the linear MR analysis, where estimation via summary level data is also possible, as sensitivity analysis we repeated the analysis using summary-data-based method to test the robustness of the results to horizontal pleiotropy. We included five summary-data-based methods, including inverse variance weighted (IVW), MR-Egger, weighted median, weighted mode, and MR-PRESSO. Different methods made varying assumptions about horizontal pleiotropy, and consistent results across different methods suggest that the causal inference was more credible.^26^ Further details and information on additional sensitivity analyses is provided in Supplementary methods.

Analyses were conducted using STATA version 17.0 (StataCorp LP, College Station, Texas, USA) and R (version 4.2.0). ^27, 28^

## Results

Up to 278,093 individuals were included in the analysis. The mean alcohol consumption was 18.20 grams per day (SD 18.81). Male participants and those who smoked, engaged in intense physical activity, had completed NVQ/CSE/A-levels or a degree/professional education, or had the highest TDI, also had slightly higher average alcohol consumption compared to others. Participants who reported ‘fair’ self-rated health and those who did not have chronic illnesses consumed more alcohol compared to others (Table 1). The mortality rate varied across the socio-demographic, health, and lifestyle characteristics (Supplementary Table S3). Alcohol GRS was not associated with confounders except for smoking and TDI (Supplementary Table S4).

**Figure 1:**
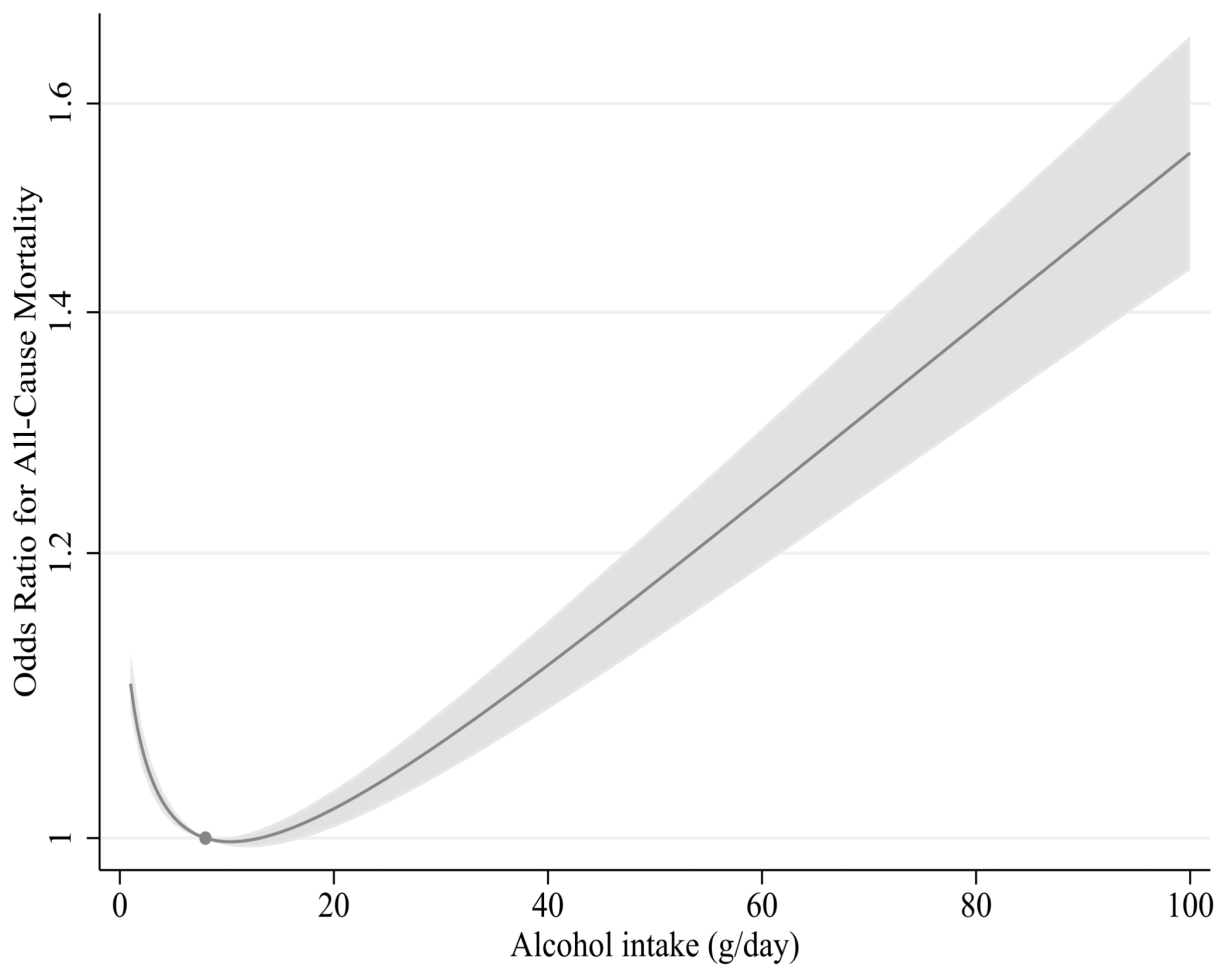
Nonlinear relationship between reported average daily alcohol consumption and all-cause mortality. The dot in the figure represents the reference point (8 grams per day), and the shaded region is the 95% confidence interval. The analyses are adjusted for sex, age, assessment centre, birth location, educational status, TDI, BMI, physical activity, and smoking.

Figure 1 illustrates a “J” shaped association between the reported alcohol consumption and all-cause mortality. Those who did not drink or drank in small amounts had a higher risk of premature death compared to moderate drinkers, and heavy drinkers had an even greater risk. The curved pattern was consistent across CVD, cancer, digestive, and respiratory mortality. A similar pattern was observed when limiting the analysis to deaths occurring before the onset of the COVID-19 pandemic (up until January 1, 2020) (Supplementary Figure S4 and Supplementary Table S5). There was no statistical association between alcohol intake and COVID-19 mortality (P-value = 0·66 and P_non-linearity_ = 0.77). Sensitivity analyses removing the BMI adjustment provided similar results (Supplementary Table S5). We also conducted sensitivity analyses stratifying by age and self-reported health which provided support for some influences by selection bias and reverse causality in the conventional observational analyses (Supplementary Table S6).

**Figure 2:**
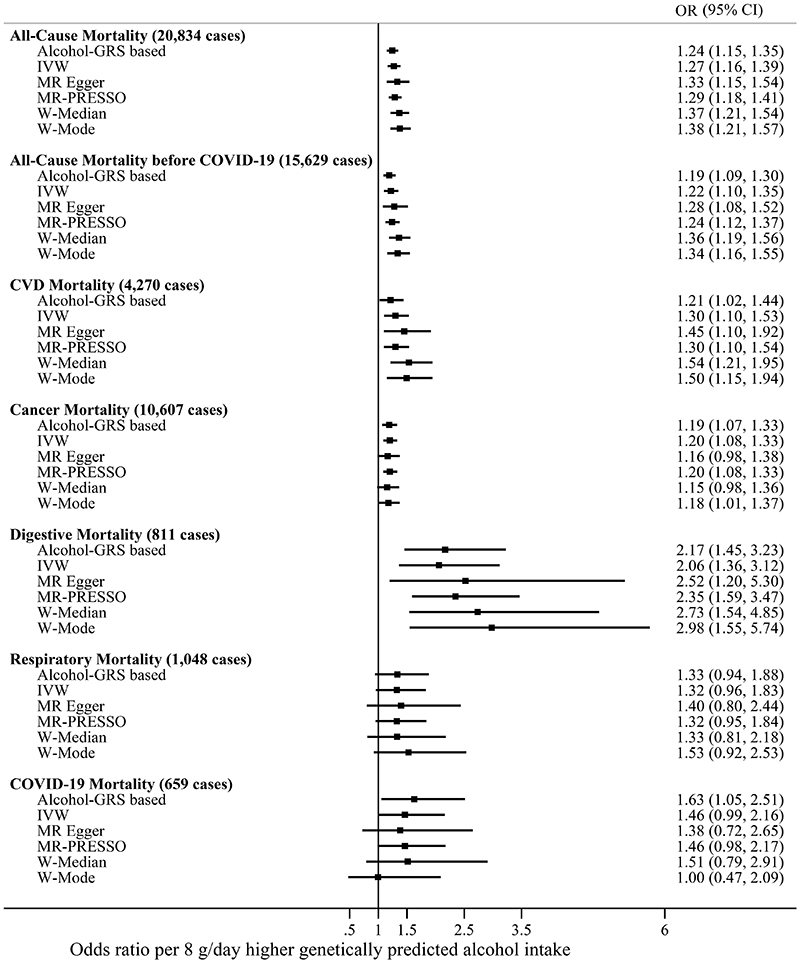
Linear MR analyses of alcohol consumption with all-cause- and cause-specific mortality. The point estimates are represented by squares and the 95% confidence intervals by horizontal bars. Adjustments were made for age, sex, assessment centre, birth location, SNP array, and the top 40 genetic principal components. IVW - inverse-variance weighted MR; W-Median - weighted median MR; W-Mode - weighted mode MR. g/day – grams per day

Figure 2 shows the findings from the linear MR analysis on genetically predicted alcohol consumption and mortality. The results support a positive relationship between alcohol consumption and all-cause mortality (OR by 8 grams higher daily alcohol intake 1·27, 95% CI 1·16–1·39), CVD mortality (1·30, 1·10–1·53), cancer mortality (1·20, 1·08–1·33), and digestive mortality (2·06, 1·36–3·12). The associations between genetically predicted alcohol consumption and respiratory (1·32, 0·96–1·83) and COVID-19 disease mortality (1·46, 0·99–2·16) were imprecise. Findings from analyses using the GRS-based and summary-data-based approaches were generally consistent for all outcomes (Figure 2). Adjustment for smoking and TDI did not affect the results (Supplementary Table S7).

**Figure 3:**
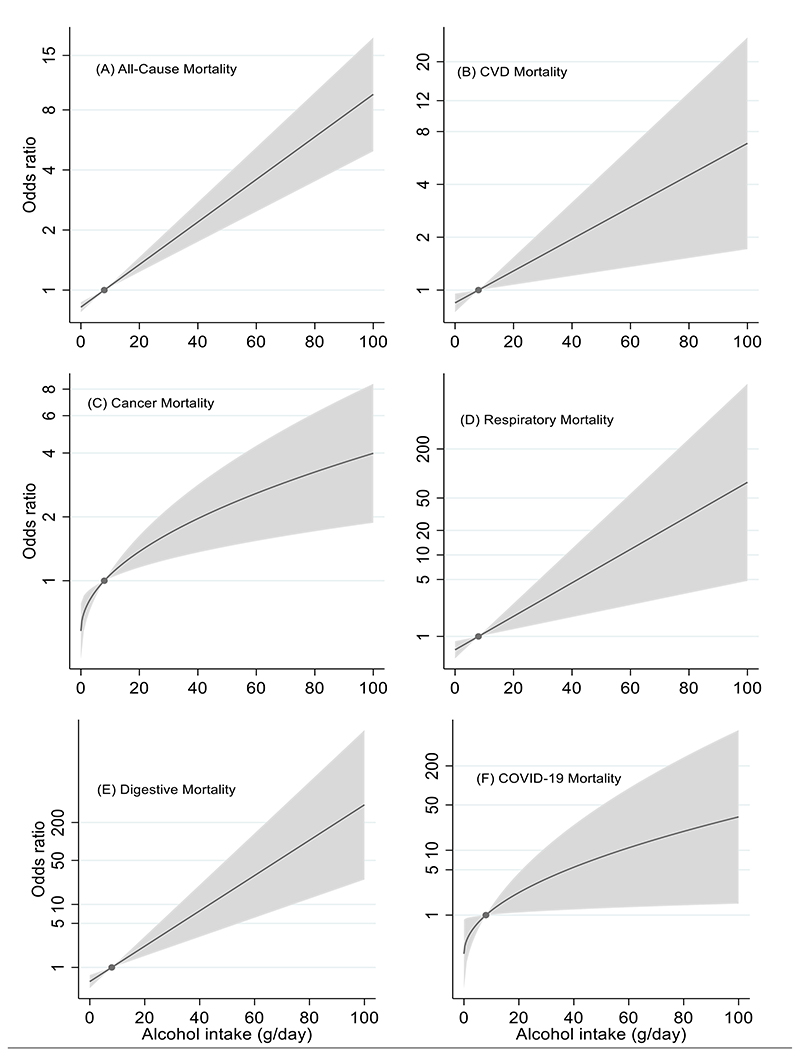
Genetically predicted alcohol intake and mortality by the mean level of alcohol intake from nonlinear MR analyses. (A) All-cause mortality; (B) CVD mortality; (C) Cancer mortality; (D) Respiratory mortality; (E) Digestive mortality (F) COVID-19 mortality in the UK Biobank. For all models P_nonlinearity_ ≥ 0.21 suggesting the linear model had the best fit. The odds ratio with the 95% confidence intervals for genetically predicted alcohol intake falls in the shaded area, and the dot represent the reference point (8 grams per day). Associations were adjusted for age, sex, assessment centre, birth location, SNP array, and the top 40 genetic principal components. CVD-cardiovascular disease

Nonlinear MR analyses did not provide any evidence to support the J-shaped association between alcohol intake and mortality suggested in the conventional observational analysis. In contrast, the doubly-ranked method confirmed the linear association, revealing that higher genetically predicted alcohol consumption was associated with increased mortality from all-causes, including respiratory and COVID-19 diseases (Figure 3). Notably, the association between alcohol intake and all-cause mortality remained similar when restricted to deaths before the COVID-19 pandemic (Supplementary Figure S5).

Genetic effects on alcohol intake were stronger in strata with higher mean alcohol intake (Supplementary Figure S6), supporting the use of doubly-ranked MR approach for the non-linear analyses. Further details for instrument validation, stratum-specific estimates and sensitivity analyses, is provided in the Supplementary text pages 2-6, Supplementary Figures S7-S11 and Supplementary Table S8.

## Discussion

The proposed benefits of low to moderate alcohol intakes^6, 29^ have been a source of long-standing debate and controversy. We investigated the association between alcohol intake and mortality using a genetic approach, confirming the risks associated with heavy consumption but no evidence to support any benefits for moderate intakes. Indeed, we observed linear increases in the risk of premature mortality across different contributing causes, suggesting that reduction in the amount of alcohol consumed is likely to be beneficial regardless of the level of current intake.

Conventional observational analyses often show a “J-shaped” relationship between alcohol consumption and mortality,^5, 11^ which was also observed in our analyses. Previous studies have focussed on cause specific mortality risk,^11^ for example finding evidence for a lower risk of CVD and cancer mortality in light or moderate drinkers compared to non-drinkers. However, benefits have not been consistently observed^30^ and many have argued that the impact of light to moderate alcohol consumption on premature death is likely to be driven by biases such as reverse causality, confounding, and selection bias. Supporting this notion, meta-analysis of 87 studies found that low-volume drinking may have protective effects, but only when selection biases and study quality are not taken into account.^31^ Here we demonstrate that the alcohol-mortality relationship differs between younger and older individuals, and is strongly affected by adjustment to potential confounders and indicators of poor health, supporting the role of selection bias and reverse causality. We used a MR approach which can, at least to some extent, address these biases. All but one of the previous MR studies have assumed linear effects, using an analytical approach that would not have been able to detect protective effects from modest alcohol intake, should they exist. In the earlier non-linear MR study the authors observed a convex association,^16^ but the analytical method used assumed a constant association between the genetic instrument and alcohol intake, which we now show is strongly violated in this context (Supplementary Figure S6). In our analyses using the doubly-ranked approach^25^ we can relax this assumption, and while we may not be able to fully discount possible non-linearity, results obtained across all our analyses are more compatible with a linear effect. This was also suggested by an earlier study that approximated alcohol intakes based on regional location, and showed that the alcohol related cardiovascular risks are similar regardless of average level of intake.^15^

Multiple mechanisms may contribute to the harmful effects of alcohol on cells and tissues, and the mediating pathways may differ between different causes of death. One mechanism with possible broad impact involves the impact of alcohol metabolism on the body’s systemic oxidative and inflammatory state, which generates toxic intermediates and metabolic stress.^32–34^ Additionally, alcohol exerts a direct effect on cellular components, which alters their biological function.^32^ Consequently, alcohol intake may induce chronic inflammation and metabolic changes, which can increase the risk of several types of deaths. Particularly relevant for cardiovascular health, drinking alcohol can raise blood pressure,^35^ and if consumed excessively, these effects may be aggravated by inflammation and harm to the heart muscles.^36^ Contrary to popular belief that moderate alcohol consumption may enhance heart health by increasing HDL cholesterol and adiponectin levels while lowering fibrinogen levels,^37^ studies have shown that alcohol intake can have the opposite effect. It can raise LDL cholesterol levels and decrease HDL cholesterol levels in the bloodstream.^38^ Furthermore, multiple MR and RCT studies have found no evidence linking elevated levels of HDL cholesterol or fibrinogen to a reduced risk of CVD.^39^ Acetaldehyde, the toxic by-product of alcohol metabolism, can damage DNA and other cellular components, contributing to cancer mortality. Moreover, ethanol consumption can suppress the immune system, alter hormonal and chemical levels, and facilitate the development of cancer in various organs.^40, 41^ Alcohol can also impair liver function, increase inflammation in the digestive tract, interfere with nutrient absorption, lead to deficiencies, and cause digestive problems such as acid reflux and heartburn, or irritate and damage the digestive tract lining.^42^ Lastly, alcohol can disrupt the functioning of airway cilia and alveolar macrophages, induce mast cells, impair the immune system, and cause inflammation in the respiratory tract.^43, 44^

Strengths of our study include the large sample and the genetic analysis approach which allowed us to explore the association between moderate alcohol consumption and mortality risk largely avoiding influences from reverse causality and confounding which commonly bias conventional epidemiological analyses of observational studies. Our approach also allows for the exploration of causal associations at any level of intake without subjecting participants to potential harm, which is unlikely to apply for any RCT conducted in this context. To ensure the consistency of our estimates, we utilized multiple linear MR methods. To the best of our understanding, this is the first study of its kind to employ the doubly-ranked method for non-linear MR analysis to examine multiple cause-specific mortality types. This new method allows us to overcome the assumption that the association between the genetic instrument (here, alcohol GRS) remains constant across differing levels of alcohol consumption, which may be violated and led to a bias in previous non-linear MR studies.^17, 25^ However, our study also has some limitations. As information on alcohol consumption is typically obtained through self-report, calculated amounts may not be entirely accurate. This may introduce bias into the stratification of individuals into different strata and on weights used to calculate the genetically predicted intakes. Although we utilized several MR methods to address issues of pleiotropy and weak instrument bias, we cannot fully discount related effects. Indeed, the alcohol GRS was associated with TDI and smoking, but it is uncertain whether this reflects pleiotropy or downstream effects of higher alcohol intakes. Adjustment for TDI and smoking did not materially affect results. Furthermore, while there is some evidence supporting the relevance of our findings to other ethnic groups,^11^ we restricted the sample to white-British individuals, which reduces the generalizability of our results to individuals from other ancestries. Like all MR studies, our use of genetic instruments to approximate average effects over the life-course may not fully capture the true biological association between alcohol consumption and mortality risk, which could vary in shape and strength at different life-stages and be more complex than reflected in our study. MR cannot eliminate bias from competing risks, hence, causal estimates for cause-specific mortalities may be biased towards the null, especially for conditions that typically affect people of older age. The UK Biobank, despite its large sample size, has only a 5% response rate and may not be representative of the general public in the UK. While selection bias is likely to affect findings from linear MR studies less compared to other designs,^45^ it is difficult to determine the extent to which selection may have affected our MR analysis. In particular, differential selection bias can induce genetic associations within strata of the population, even for the doubly-ranked method.^46^ We have adjusted for age and sex, as these are the strongest predictors of selection, which should mitigate the influence of differential selection based on these variables.^47^ Nevertheless, some residual bias due to different selection cannot be ruled out.

Genetic evidence strongly suggests that as alcohol consumption increases, there is a linear increase in the risk of premature death, including from specific causes such as CVD, cancer, and digestive illness, with no evidence for any protection by modest intakes. While the greatest mortality risks are associated with heavy drinking, public health initiatives should prioritize efforts to reduce alcohol intakes at all levels of consumption. Re-evaluation of current public policies regarding drinking guidelines may be warranted.

## Supplementary Material

Supplementary material

## Figures and Tables

**Table 1:** Alcohol consumption by baseline characteristics in UK Biobank.

	N (%)	Alcohol intake (g/day)
		Mean (SD)
**Total**	278093 (100)	18·20 (18·81)
**Age (years)**		
<65	222626 (80·05)	18·57 (19·05)
≥65	55467 (19·95)	16·69 (17·74)
P-value^a^		8·17E-189
**Sex**		
Male	135319 (48·66)	24·51 (22·05)
Female	142774 (51·34)	12·21 (12·45)
P-value^a^		<1·0E-300
**BMI (kg/m2)**		
<18.5	1341 (0·48)	13·49 (18·74)
(18·5-25)	91655 (32·96)	16·11 (16·51)
(25-30)	120519 (43·34)	19·50 (19·1)
≥30	63722 (22·91)	18·88 (20·95)
Missing	856 (0·31)	14·74 (19·91)
P-value^a^		9·45E-60
**Smoking **		
Non-smokers	148824 (53·52)	14·65 (15·36)
Ex-smokers	100892 (36·28)	21·27 (19·66)
Smokers^b^	7390 (2·66)	25·27(21·34)
Cigars/pipes	1670 (0·6)	32·12 (27·54)
≤1 to 15 cigs/day	10966 (3·94)	22·85 (23·97)
>15 cigs/day	7423 (2·67)	30·62 (33·23)
Missing	928 (0·33)	16·94 (17·07)
P-value ^a^		<1·0E-300
**Physical activity**		
Light	81647 (29·36)	17·21 (18·68)
Moderate	136225 (48·99)	18·26 (17·98)
High	54336 (19·54)	19·59 (20·35)
Missing	5885 (2·12)	17·44 (23·27)
P-value^a^		4·61E-53
**Education**		
Degree/professional	131960 (47·45)	18·11 (17·38)
NVQ/CSE/A-levels	98291 (35·34)	18·74 (19·73)
None of the above	45694 (16·43)	17·42 (20·69)
Missing	2148 (0·77)	14·99 (17·18)
P-value^a^		3·61E-44
**Townsend index**		
Quartile 1(least deprived)	70647 (25·4)	17·75 (16·88)
Quartile 2	70429 (25·33)	17·70 (17·44)
Quartile 3	69692 (25·06)	18·13 (18·71)
Quartile 4(most deprived)	66993 (24·09)	19·27 (21·92)
Missing	332 (0·12)	17·96 (21·41)
P-value^a^		2·95E-41
**Self-rated health**		
Excellent	47534 (17·09)	17·68 (16·01)
Good	163846 (58·92)	18·06 (17·83)
Fair	55063 (19·8)	19·07 (21·76)
Poor	10770 (3·87)	18·04 (26·4)
Missing	880 (0·32)	18·85 (23·34)
P-value^a^		6·79E-17
**Long-term illness**		
No	186033 (66·9)	18·50 (18·04)
Yes	86100 (30·96)	17·60 (20·31)
Missing	5960 (2·14)	17·25 (19·68)
P-value^a^		2·21E-88

g/day – grams per day; SD – standard deviation; BMI (Kg/m^2^) - body mass index (kilogram per meter square); CSE - Certificate of Secondary Education; NVQ - National Vocational Qualification;

a P-values are from linear regression test with missing category excluded. We included age, sex, assessment centre, for adjustment;

b Smokers without information on types of tobacco that they smoke.

## Data Availability

The data used in this research can be accessed by researchers who meet the required criteria and obtain necessary approvals from the UK Biobank access management committee at the University of Oxford. More information about accessing the research database can be found on the UK Biobank website at https://www.ukbiobank.ac.uk/.

## Key Messages

- Genetically predicted alcohol intake was associated with an increased risk of premature death, including all leading underlying causes.
- Genetic analysis did not provide evidence for a non-linear association, failing to support observational studies suggesting benefits by modest alcohol intakes.
- While the greatest mortality risks are seen with high intakes, lowering guidelines for safe alcohol consumption would benefit public health, as even moderate intake poses a risk.
