# Supplementary material for: Alcohol consumption and the risk of all-cause and cause-specific mortality—a linear and nonlinear Mendelian randomization study
Source: Int J Epidemiol. 2024 Mar 20;53(2):dyae046. doi: 10.1093/ije/dyae046 (PMC10951973; doi:10.1093/ije/dyae046)
Supplement: Supplementary file 1 [file dyae046_supplementary_data.docx]

SUPPLEMENTARY MATERIALS 

**Contents**

Supplementary methods	2

Alcohol consumption measures	2

Genetic variants, genetic risk score, and Instruments Validation	2

Adjustments and covariate definitions	3

Summary-data-based linear MR methods	4

Non-linear Mendelian randomization method	4

Sensitivity analyses to investigate reverse causality and selection bias in the conventional observational analyses.	6

Supplementary Table S1.  Definitions of outcomes considered.	7

Supplementary Table S2. Genome-wide significant alcohol consumption variants used for the genetic instruments.	7

Supplementary Table S3: All-Cause and Cause-specific- mortality by baseline characteristics in the UK Biobank.	9

Supplementary Table S4: Association of Alcohol GRS with baseline characteristics in the UK Biobank.	10

Supplementary Table S5. Conventional nonlinear observational analysis by different covariates.	10

Supplementary Table S6. The association between alcohol intake and all-cause mortality stratified by age and self-rated health status.	11

Supplementary Table S7: MR estimates from linear MR analyses further adjusted with smoking and TDI.	11

Supplementary Table S8. Nonlinear MR test for nonlinearity across the varying number of strata.	12

Supplementary Figure S1. Diagram for Mendelian randomisation  assumption.	12

Supplementary Figure S2: Participants flow chart of study.	13

Supplementary Figure S3. Schematic presentation of the analytical approach.	14

Supplementary Figure S4. Conventional nonlinear observational association between alcohol intake and mortality	15

Supplementary Figure S5: The doubly-ranked method for nonlinear MR estimates of all-cause mortality before onset of covid-19 by the average level of alcohol intake.	16

Supplementary Figure S6. The estimated genetic association with alcohol consumption at each stratum.	16

Supplementary Figure S7. The distribution of alcohol GRS across the strata.	17

Supplementary Figure S8. The distribution of alcohol consumption (g/day) across the strata.	17

Supplementary Figure S9: Association between alcohol-GRS and mortality risk across the strata.	21

Supplementary Figure S10. Local average causal effect (LACE) estimates across the strata.	24

Supplementary Figure S11.  LACE estimates of mortality against the mean level of alcohol intake in each stratum.	28

References	29

## Supplementary methods  

## Alcohol consumption measures

In the UK Biobank baseline survey conducted between 2006 and 2010, information on alcohol consumption was collected from participants through a touchscreen questionnaire. Those who drank alcohol at least once a week were asked to report their average weekly intake, while those who drank less frequently were asked to report their average monthly intake, for different types of beverages including red wine, white wine or champagne, beer/cider (such as bitter, lager, stout, ale, Guinness), spirits or liqueurs (such as whisky, gin, rum, vodka, brandy), fortified wine (include drinks such as sherry, port, vermouth), and other alcoholic drinks (such as alcopops). Pictures demonstrating a single serving size for each type of drink were provided alongside the questions. Participants were asked to report the number of glasses they consumed and were provided guidance on how many glasses are typically in a standard bottle. (refer to [https://biobank.ndph.ox.ac.uk/ukb/ukb/docs/TouchscreenQuestionsMainFinal.pdf](https://biobank.ndph.ox.ac.uk/ukb/ukb/docs/TouchscreenQuestionsMainFinal.pdf) for further details).

Initially, we computed the total amount of alcoholic drinks per person by multiplying the number of glasses of each beverage type reported by the participant. This calculation was done for all beverage types. Next, we determined the intake of pure alcohol, in grams, for each beverage type by multiplying the volume of alcoholic drinks with the alcohol by volume (ABV) and ethanol density. If the participant reported their alcohol consumption on a weekly basis, we converted it to daily consumption by dividing it by seven. For participants who provided information on a monthly basis, we estimated their weekly consumption by dividing the total volume of drinks by 4.3 and then dividing this value by 7 to obtain the daily amount. The total daily alcohol intake in grams was computed by adding the amounts of each beverage consumed, considering both weekly and monthly consumption, and then used for further analysis. 

The reason why we transformed the self-reported alcohol intake into the corresponding amount of pure ethanol consumed in grams is to enable international comparisons. In the United Kingdom, one unit of alcohol corresponds to eight grams of pure ethanol.^1^ To be included in our analysis, participants were required to have complete information on their alcohol consumption. It should be noted that self-reported alcohol intake may not be entirely accurate due to possible reporting errors.

## Genetic variants, genetic risk score, and Instruments Validation

Genotyping in the UK biobank was performed using the Affymetrix UK BiLEVE Axiom array for 50,000 participants and the Affymetrix UK Biobank axiom array for 450,000 participants. Imputation was performed using reference panels from the Haplotype Reference Consortium, UK10K, and 1000 Genomes. The UK Biobank central team performed genotyping, imputation, and related quality control, with the complete methodology described elsewhere.^2^ 

We identified genetic variants associated with alcohol consumption based on the genome-wide association meta-analysis performed by GWAS and Sequencing Consortium of Alcohol and Nicotine Use (GSCAN). (see  [https://www.ncbi.nlm.nih.gov/projects/gap/cgi-bin/study.cgi?study_id=phs001809.v1.p1](https://www.ncbi.nlm.nih.gov/projects/gap/cgi-bin/study.cgi?study_id=phs001809.v1.p1)). In this meta-analysis, 99 conditionally independent genetic variants were associated with alcohol consumption at the genome-wide significant level.^3^ The meta-analysis included 941,280 individuals of European descent, with 124,590 of them (13·2%) being from the UK Biobank cohort, as also previously mentioned^4^ the sample overlap bias^5^ is insignificant. After removing five SNPs that had a directionally inconsistent association with alcohol consumption in the UK Biobank, 94 independent SNPs were used to construct genetic risk score (GRS) for alcohol consumption, which is a summary measure that reflects the genetic predisposition of an individual to alcohol intake. 

The GRS of an individual was calculated using 94 SNPs ^3^, each coded as 0, 1, or 2, reflecting the number of alcohol-intake-increasing alleles. To construct it, we first computed the weighted average of the number of alcohol-intake-increasing alleles for an individual, and then multiplied this by the number of available SNPs. The weight assigned to each SNP was the effect estimate of its association with alcohol intake, taken from the GSCAN GWAS.^3^

The instrument’s validity is crucial in MR analysis. For a genetic instrument to be considered valid, it must fulfill the three key assumptions^6^ presented in Supplementary Figure S1. The first assumption is typically the strength of association between the genetic instrument and the exposure (in this case alcohol intake). An F-statistic of greater than ten is recommended to minimise weak instrument bias ^7^. In our study, the GRS-alcohol intake association in the analytical sample has an F-statistic of 1942·23, providing assurance that our analysis is unlikely to have been affected by the weak instrument bias. The second assumption of MR is that genetic variations do not exhibit any association with confounders of the exposure and outcome of interest. To gauge the validity of this assumption, we tested and reported the associations between the genetic instrument and any potential confounding factors. As part of our sensitivity analysis, we also incorporated these factors as covariates when examining the causal association between alcohol consumption and mortality.

The exclusion-restriction assumption states that the genetic instrument affects the outcome only via the exposure of interest. This assumption is violated when the instrument exhibits horizontal pleiotropy, whereby it affects traits that influence the outcome independently of the exposure of interest. To gauge the robustness of our MR finding to horizontal pleiotropy, as sensitivity analysis, we re-analysed the data using several pleiotropy-robust MR approaches (see section titled summary-data-based linear MR methods). These summary-data-based approached have relatively independent assumption regarding horizontal pleiotropy. When we observe a consensus in results across different methods, we gain more confidence about our findings.

## Adjustments and covariate definitions 

Covariate selection for the adjustments was dependent upon the conceptualisation on whether a factor is likely to be a confounder in particular analysis, and this differed for observational and genetic analyses. As recommended by Haworth and colleagues, we accounted for latent population structure in the UK Biobank by including age, sex,  assessment centre, types of genotyping array, birth location and 40 genetic principal components in the adjustment for genetic analyses.^8^ We tested for the  association between the GRS and covariates included in the observational analyses (Supplementary table S4) and conducted additional sensitivity analyses including further adjustments for smoking and TDI (Supplementary Table S7).  In the conventional nonlinear observational analyses, we included traditional confounders that could distort an association between alcohol intake  and mortality (age, sex, education, assessment centre, birth location, body mass index (BMI), smoking, physical activity, and self-reported health status and long-term illness, and Townsend deprivation index (TDI). We also conducted additional sensitivity analysis by further adjusting with SNP array, and top 40 genetic principal components, to confirm lack of effect by additional controlling for covariates affecting population structure   (Supplementary Table S5).  

We obtained all the covariates at the UK biobank baseline survey. All adjustments were done using age as a continuous variable. Education was classified into three categories: vocational education/secondary education/A-levels completed, and higher education (university degree or other professional qualification) completed, and none of these. To determine BMI, the weight of an individual in kilograms is divided by the square of their height in meters. Smoking habits were classified into various categories, including non-smokers, ex-smokers, current smokers with no information on the type of tobacco, cigar/pipe smokers, and cigarette smokers who smoke less than 1–5 cigarettes per day, 6-10 cigarettes per day, 11–15 cigarettes per day, 16–20 cigarettes per day, 21–25 cigarettes per day, and more than 25 cigarettes per day. Physical activity intensity was classified as either light, moderate, or high. Self-reported health was classified as poor, fair, good, or excellent, while long-term illness was defined as either present or absent. The TDI was divided into four quartiles (1 to 4), and participants were assigned a score based on their postal code's output area (the smallest UK census area), which serves as a proxy for socio-economic status by considering factors related to employment, home ownership, car ownership, and household overcrowding. A higher TDI score indicates greater socio-economic deprivation.^9^ 

## Summary-data-based linear MR methods

We conducted summary-data-based linear MR analyses by leveraging genetic variation (SNPs) as instrumental variables obtained from the UK biobank. The SNP-mortality estimate is obtained from the total UK Biobank sample, while the SNP-alcohol intake association estimate is derived from the non-deceased individuals of the whole cohort. This is to avoid potential bias resulting from overlapping samples.^5^ 

We employed five distinct MR methods - inverse variance weighted (IVW), MR-Egger, weighted median, weighted mode, and MR-PRESSO. The IVW-MR approach provides an unbiased causal estimates when there is no presence of directional pleiotropy.^10^ If there is a directional pleiotropy, the MR-Egger is a reliable approach for causal estimate of an exposure on a disease outcome.^11, 12^ The weighted median MR approach provides unbiased estimates when at least 50% of the total weight comes from a valid instruments.^13^ The weighted mode-based method groups SNPs based on the similarity of their Wald estimates and calculates the causal effect estimate using the cluster with the highest weight.^14^ MR-PRESSO detects an outlier  and eliminate possible  pleiotropic outlier variants to provide outlier-corrected causal estimation. The estimate is similar with the IVW MR when there is not a genetic variant assumed outlier.^15^ When these complementary MR methods produce consistent causal estimates, it indicates strong evidence for a causal relationship.^6^  In our study, the MR estimates across all the methods were concordant. 

Furthermore, in our summary-data-based linear MR analyses as a sensitivity analysis, we adjusted with TDI and smoking to account potential bias and confounding effect. However, there were no substantial changes in the findings (Supplementary Table S7).

## Non-linear Mendelian randomization method

We used the non-linear Mendelian randomization approach to investigate the shape of causal association between alcohol and mortality. The non-linear MR can be viewed as an extension to the standard MR which allows the effect of the exposure on the outcome to vary by the level of exposure. More specifically, in the non-linear MR analysis, the cohort is first stratified into subgroups with different average levels of the exposure. Localized average cause effect (LACE) estimates are then computed for each stratum of the cohort, and subsequently used to piece together the exposure-outcome association curve. Computation of LACE and fitting of fractional polynomial models using LACEs across strata have been detailed in the method section in the main text.

The stratification step in the non-linear MR analysis is non-trivial - directly stratifying on the exposure can lead to collider bias, as in the MR setting, exposure is a common effect of genetic instruments and confounders (Supplementary Figure 1). Two stratification methods have been proposed to circumvent the related bias: a residual method and a doubly-ranked method. An ideal stratifier should not only reflect the distribution of exposure but also be independent of genetic instruments, enabling unbiased LACE estimates across strata with different average levels of exposure. The residual method is implemented by first regressing the exposure on the genetic instrument to obtain residuals. These ‘residual’ exposures, which remain highly correlated with the original exposure yet are now independent of the genetic instrument, are then used to stratify the population.^16^ In the doubly-ranked method, participants are first ranked into pre-strata according to their level of the genetic instrument, and are then ranked within each pre-stratum according to their level of the exposure. This exposure ranking is then used to stratify the population.^4^ By design, the exposure ranking reflects differences in exposure levels. Also, since the ranking is conditioned on the level of genetic instruments, it is independent of genetic instruments.

As with all methods, validity of stratification methods is also subject to certain assumptions. For the residual stratification method, as it relies on the linear regression model to obtain ‘residual’ exposure, this imposes a strong assumption on the effect of the genetic instrument on the exposure, and requires that the effect of the genetic instrument be linear and constant for all individuals in the population.^17^ If the assumption of linearity and homogeneity is violated, the functional relationship between the genetic instrument and the exposure is not fully broken. As a result, ‘residual’ exposure is still a collider, and hence stratification can result in collider bias^17^ and lead to biased LACE estimates and inflated type 1 error rates.^4^ In contrast to the residual method, the doubly-ranked stratification method does not make any parametric assumption on the effect of the genetic instrument; instead, it assumes that an individual's counterfactual exposure ranking is the same at all levels of the genetic instrument, which is a much weaker assumption than the linear and homogeneous assumption made by the residual method.^4^ Robustness of the doubly-ranked method to the violation of the assumption of linearity and homogeneity has been demonstrated in extensive simulation analyses across a wide range of scenarios.^4, 17^

In our non-linear MR analysis, we used the doubly-ranked method to stratify the cohort. Our choice of the stratification method is justified for two reasons: 1) Alcohol intake is a coarsened (i.e., imprecisely measured) exposure. In such scenarios, the doubly-ranked method can provide unbiased estimates, whereas using the residual method could result in substantial bias.^4^ 2) The association between GRS and alcohol intake varies strongly across strata (Supplementary Figure S6), an indication of violation of the homogeneity assumption, to which the residual method is particularly sensitive.^4, 17^

We performed several complementary/sensitivity analyses to gauge the robustness of our non-linear MR analysis. Firstly, as a sanity check, we examined the distribution of alcohol GRS and alcohol intake across strata. Supplementary Figure S7 and S8 show that strata are independent of the alcohol GRS, and a higher stratum is correlated with a higher alcohol intake on average, providing reassurance that collider bias is unlikely to have operated in this setting. Secondly, we examined whether our non-linear MR findings are dependent on the choice of numbers of strata. We found that the best-fitting model is the linear model, regardless of whether the model was based on 3, 5, 10, or 50 strata (Supplementary Table S8). Furthermore, recent analyses using BMI and vitamin D genetic scores suggest that genetic associations with sex and age can be present in strata of the population defined by the doubly-ranked method.^18^ Such associations are biologically impossible and represent differential selection bias. In our nonlinear MR analysis, we have adjusted for age and sex, since these are the strongest predictors of selection. This adjustment should mitigate the influence of differential selection based on these variables.^19^ To exhaust this possibility, we have also conducted additional analyses further adjusting for age^2, age:sex interaction, and age^2:sex interaction. This confirms no effect on our results, with the linear model remaining to be the best-fit (P_non-linearity_ = 1.0). Further, as it can be argued that there are other factors that may be related to selection bias, (e.g. socioeconomic deprivation such as indexed by Townsend deprivation index (TDI) or smoking), we repeated the non-linear MR analyses adjusting for smoking and TDI, confirming that adjustment for these factors does not change our results (P_non-linearity _= 1.0).

In our nonlinear MR analyses, there were no observed patterns of relationship between alcohol consumption GRS and all-cause and cause-specific mortality across strata (Supplementary Figure S9). Similarly, the LACE estimates across the strata (Supplementary Figure S10) and against the average levels of alcohol intake in each stratum (Supplementary Figure S11) did not show any discernible pattern. 

## Sensitivity analyses to investigate reverse causality and selection bias in the conventional observational analyses.

We conducted subgroup analyses by age (<50 versus ≥50 years) and self-reported health status (excellent versus poor) to explore the potential influences of selection bias and reverse causality on the phenotypic alcohol-mortality association. In Supplementary Table S6 we show that adjustment for lifestyle and health related covariates notably attenuated the apparently higher odds of mortality seen for non-drinkers comparted to light drinkers (OR 1.61 vs. 1.27 after basic and full adjustment), while changes in adjustment had relatively smaller effect for the estimates seen with heavy consumption (1.33 vs. 1.21).  This supports a notion that the observed J-shaped association is in part due to confounding and reverse causality. We also conducted analyses stratified by age (<50 years vs. ≥50 years) and self-reported health status (excellent vs. poor ). There, we observed no evidence for a J-shaped association in the younger participants while evidence for non-linearity remained in the older group, potentially supporting some bias by selection. However, stratification by self-reported health did not notably affect the shape of the association, with evidence for non-linearity seen both for those reporting poor as well as excellent health. However, depending on the analytical approach, statistical evidence for non-linearity was weaker for those reporting excellent compared to poor health. These findings are broadly consistent with expectations under the influence of selection bias and reverse causality as suggested by the reviewer, and are also in line with the finding from a meta-analysis of 87 studies, where the ‘protective effect’ associated with low-volume drinking is only observed when selection biases and study quality are not taken into account.^20^

 **Statistical software’s and packages used for the analyses.** 

We used STATA version 17.0 (StataCorp LP, College Station, Texas, USA) for GRS-based linear MR analysis, and R (version 4.2.0) for the summary-data-based linear MR analysis (TwoSampleMR package)^21^ and non-linear MR analysis (SUMnlmr package).^22^

## Supplementary Table S1.  Definitions of outcomes considered.

| **Endpoint** | **ICD-10 codes** |
| --- | --- |
| CVD mortality | I00 to I89 |
| Cancer mortality | C00 to D48 |
| Respiratory diseases mortality | J09 to J18, J20 to J22, and J40–J47 |
| Digestive diseases mortality | K00 to K95 |
| COVID-19 disease mortality | U07·1, U07·2 |

## Supplementary Table S2. Genome-wide significant alcohol consumption variants used for the genetic instruments.

|  |  |  |  |  |  |  | **GSCAN Meta-analysis**^**1**^ |  |  | **UK Biobank**^**2**^ |  |  |
| --- | --- | --- | --- | --- | --- | --- | --- | --- | --- | --- | --- | --- |
| **SNP** | **CHR** | **BP** | **Gene** | **A1** | **A2** | **A1F** | **Beta** | **SE** | **P** | **Beta** | **SE** | **P** |
| rs10753661 | 1 | 165119792 | Intergenic | G | A | 0·316 | 0·0086 | 0·0016 | 3·8E-08 | 0·1758488 | 0·0511013 | 5·79E-04 |
| rs12088813 | 1 | 66407700 | Intron:PDE4B | A | C | 0·733 | 0·0093 | 0·0016 | 1·6E-08 | 0·1566171 | 0·0531379 | 3·21E-03 |
| rs28680958 | 1 | 173848808 | Intron:ZBTB37 | G | A | 0·783 | 0·0110 | 0·0018 | 5·1E-10 | 0·2675408 | 0·0575084 | 3·29E-06 |
| rs5024204 | 1 | 71491890 | Intron:PTGER3 | T | A | 0·278 | 0·0097 | 0·0016 | 2·6E-09 | 0·1536786 | 0·0535243 | 4·09E-03 |
| rs58107686 | 1 | 33837334 | Intron:PHC2 | C | A | 0·672 | 0·0097 | 0·0016 | 7·8E-10 | 0·1442787 | 0·0503214 | 4·14E-03 |
| rs705687 | 1 | 4548453 | Intergenic | A | G | 0·215 | 0·0109 | 0·0018 | 8·2E-10 | 0·2258396 | 0·0567171 | 6·84E-05 |
| rs823114 | 1 | 205719532 | Intergenic | A | G | 0·553 | 0·0088 | 0·0015 | 2·3E-09 | 0·0825498 | 0·0474233 | 8·17E-02 |
| rs1004787 | 2 | 45159091 | Intron:LINC01833 | A | G | 0·551 | 0·0084 | 0·0015 | 8·4E-09 | 0·3953034 | 0·0479605 | 1·70E-16 |
| rs11692435 | 2 | 98275354 | Nonsynonymous:ACTR1B | A | G | 0·085 | 0·0174 | 0·0026 | 2·5E-11 | 0·4736314 | 0·0913206 | 2·14E-07 |
| rs1260326 | 2 | 27730940 | Nonsynonymous:GCKR | C | T | 0·601 | 0·0209 | 0·0015 | 8·1E-45 | 0·5773892 | 0·0484119 | 8·76E-33 |
| rs13024996 | 2 | 144225215 | Intron:ARHGAP15 | C | A | 0·636 | 0·0109 | 0·0015 | 5·7E-13 | 0·2619200 | 0·0488855 | 8·43E-08 |
| rs13032049 | 2 | 63581507 | Intron:WDPCP | G | A | 0·283 | 0·0102 | 0·0016 | 3·0E-10 | 0·1965810 | 0·0529486 | 2·05E-04 |
| rs13383034 | 2 | 45155276 | Intron:LINC01833 | T | C | 0·329 | 0·0149 | 0·0016 | 6·3E-22 | 0·3927672 | 0·0515973 | 2·70E-14 |
| rs56337305 | 2 | 225475560 | Intergenic | T | C | 0·617 | 0·0096 | 0·0015 | 1·6E-10 | 0·1718381 | 0·0494119 | 5·06E-04 |
| rs72859280 | 2 | 147956293 | Intergenic | T | G | 0·036 | 0·0229 | 0·0039 | 4·4E-09 | 0·1826883 | 0·1305685 | 1·62E-01 |
| rs77165542 | 2 | 430975 | Intergenic | C | T | 0·965 | 0·0260 | 0·0040 | 5·6E-11 | 0·2951151 | 0·1309877 | 2·43E-02 |
| rs828867 | 2 | 74334462 | Utr3:TET3 | A | G | 0·545 | 0·0088 | 0·0015 | 2·2E-09 | 0·1719034 | 0·0491616 | 4·71E-04 |
| rs13066454 | 3 | 93994255 | Intergenic | C | T | 0·602 | 0·0088 | 0·0015 | 4·1E-09 | 0·1476003 | 0·0483799 | 2·28E-03 |
| rs13094887 | 3 | 70968431 | Intergenic | A | T | 0·699 | 0·0103 | 0·0016 | 8·6E-11 | 0·1649271 | 0·0519106 | 1·49E-03 |
| rs2011092 | 3 | 141124607 | Intron:ZBTB38 | T | C | 0·661 | 0·0089 | 0·0015 | 7·4E-09 | 0·0014974 | 0·0497293 | 9·76E-01 |
| rs60654199 | 3 | 141267295 | Intron:RASA2 | C | A | 0·937 | 0·0167 | 0·0030 | 2·9E-08 | 0·2712640 | 0·0950069 | 4·30E-03 |
| rs62250685 | 3 | 85457240 | Intron:CADM2 | A | G | 0·386 | 0·0144 | 0·0015 | 1·1E-21 | 0·2227364 | 0·0486880 | 4·77E-06 |
| rs6787172 | 3 | 158187811 | Intron:RSRC1 | T | G | 0·446 | 0·0080 | 0·0015 | 4·3E-08 | 0·1703228 | 0·0476835 | 3·54E-04 |
| rs9838144 | 3 | 131576287 | Intron:CPNE4 | G | C | 0·791 | 0·0100 | 0·0018 | 2·7E-08 | 0·1860230 | 0·0594528 | 1·75E-03 |
| rs10004020 | 4 | 152968372 | Intergenic | A | G | 0·72 | 0·0091 | 0·0016 | 2·4E-08 | 0·1630148 | 0·0526820 | 1·97E-03 |
| rs10028756 | 4 | 100254520 | Intergenic | G | A | 0·871 | 0·0186 | 0·0022 | 1·2E-17 | 0·4021978 | 0·0705262 | 1·18E-08 |
| rs11940694 | 4 | 39414993 | Intron:KLB | G | A | 0·597 | 0·0259 | 0·0015 | 3·0E-68 | 0·5103061 | 0·0492362 | 3·64E-25 |
| rs1229984 | 4 | 100239319 | Nonsynonymous:ADH1B | C | T | 0·963 | 0·1505 | 0·0039 | <2·2e-308 | 3·8195345 | 0·1632210 | 0·00E+00 |
| rs12499107 | 4 | 99678691 | Intergenic | G | A | 0·131 | 0·0127 | 0·0022 | 4·5E-09 | 0·1902300 | 0·0699748 | 6·56E-03 |
| rs12651313 | 4 | 171086393 | Intergenic | C | G | 0·557 | 0·0086 | 0·0015 | 3·8E-09 | 0·1947640 | 0·0478200 | 4·65E-05 |
| rs13107325 | 4 | 103188709 | Nonsynonymous:SLC39A8 | C | T | 0·928 | 0·0275 | 0·0028 | 1·5E-22 | 0·7563803 | 0·0902627 | 5·33E-17 |
| rs144198753 | 4 | 99713350 | Intergenic | C | T | 0·984 | 0·0418 | 0·0059 | 1·4E-12 | 3·7331092 | 0·2769494 | 0·00E+00 |
| rs2165670 | 4 | 100286085 | Intergenic | A | G | 0·106 | 0·0231 | 0·0024 | 1·7E-22 | 0·5813457 | 0·0780850 | 9·72E-14 |
| rs35538052 | 4 | 39418965 | Intron:KLB | G | A | 0·621 | 0·0085 | 0·0015 | 1·4E-08 | 0·5078633 | 0·0494650 | 1·00E-24 |
| rs36052336 | 4 | 100273594 | Intron:ADH1C | A | G | 0·939 | 0·0184 | 0·0030 | 1·2E-09 | 0·1971731 | 0·0986212 | 4·56E-02 |
| rs3748034 | 4 | 3446091 | Nonsynonymous:HGFAC | G | T | 0·857 | 0·0117 | 0·0021 | 1·7E-08 | 0·1773473 | 0·0678396 | 8·94E-03 |
| rs4501255 | 4 | 42151306 | Intron:BEND4 | G | C | 0·235 | 0·0107 | 0·0017 | 4·8E-10 | 0·1702274 | 0·0555719 | 2·19E-03 |
| rs4690727 | 4 | 143648579 | Intron:INPP4B | G | C | 0·718 | 0·0108 | 0·0016 | 2·4E-11 | 0·2457692 | 0·0530781 | 3·65E-06 |
| rs4699791 | 4 | 101243023 | Intergenic | A | G | 0·096 | 0·0186 | 0·0025 | 6·6E-14 | 0·0498837 | 0·0807275 | 5·37E-01 |
| rs561222871 | 4 | 100260679 | Intron:ADH1C | C | T | 0·953 | 0·0388 | 0·0036 | 6·6E-27 | 1·2545691 | 2·4901154 | 6·14E-01 |
| rs79139602 | 4 | 100444363 | Intron:C4orf17 | T | A | 0·021 | 0·0603 | 0·0051 | 1·8E-32 | 0·2067894 | 0·1685861 | 2·20E-01 |
| rs11739827 | 5 | 166803321 | Intron:TENM2 | G | T | 0·549 | 0·0084 | 0·0015 | 1·2E-08 | 0·1744965 | 0·0482562 | 2·99E-04 |
| rs12655091 | 5 | 144412335 | Intergenic | G | A | 0·47 | 0·0083 | 0·0015 | 1·3E-08 | 0·1495702 | 0·0474073 | 1·61E-03 |
| rs4916723 | 5 | 87854395 | Intron:LINC00461 | A | C | 0·584 | 0·0100 | 0·0015 | 1·7E-11 | 0·1798325 | 0·0490093 | 2·43E-04 |
| rs55872084 | 5 | 155902003 | Intron:SGCD | T | G | 0·235 | 0·0100 | 0·0017 | 6·3E-09 | 0·0843162 | 0·0553498 | 1·28E-01 |
| rs10085696 | 7 | 69783020 | Intron:AUTS2 | A | G | 0·814 | 0·0114 | 0·0019 | 1·1E-09 | 0·2178989 | 0·0610955 | 3·62E-04 |
| rs10236149 | 7 | 98977515 | Intron:ARPC1B | A | G | 0·877 | 0·0135 | 0·0022 | 1·2E-09 | 0·2080634 | 0·0715551 | 3·64E-03 |
| rs35034355 | 7 | 103840115 | Intron:ORC5 | G | A | 0·479 | 0·0081 | 0·0015 | 2·9E-08 | 0·2112134 | 0·0473476 | 8·16E-06 |
| rs6460047 | 7 | 73042443 | Intergenic | C | T | 0·208 | 0·0116 | 0·0018 | 9·7E-11 | 0·2602887 | 0·0583749 | 8·24E-06 |
| rs6951574 | 7 | 153489744 | Intergenic | C | T | 0·458 | 0·0132 | 0·0015 | 1·6E-19 | 0·3076286 | 0·0481412 | 1·66E-10 |
| rs1217091 | 8 | 64527399 | Intergenic | C | T | 0·812 | 0·0122 | 0·0019 | 7·1E-11 | 0·1407864 | 0·0603372 | 1·96E-02 |
| rs13250583 | 8 | 20949917 | Intergenic | C | T | 0·787 | 0·0097 | 0·0018 | 4·7E-08 | 0·1664551 | 0·0579659 | 4·08E-03 |
| rs28601761 | 8 | 126500031 | Intergenic | G | C | 0·42 | 0·0091 | 0·0015 | 7·2E-10 | 0·1936661 | 0·0491790 | 8·22E-05 |
| rs10978550 | 9 | 109345993 | Intergenic | T | C | 0·794 | 0·0117 | 0·0018 | 7·2E-11 | 0·2563484 | 0·0591416 | 1·46E-05 |
| rs55932213 | 9 | 108755622 | Intergenic | G | A | 0·736 | 0·0095 | 0·0017 | 9·6E-09 | 0·2151291 | 0·0565159 | 1·41E-04 |
| rs17665139 | 10 | 125093880 | Intergenic | C | T | 0·851 | 0·0116 | 0·0020 | 1·6E-08 | 0·2048174 | 0·0659009 | 1·88E-03 |
| rs7074871 | 10 | 110507806 | Intergenic | G | A | 0·745 | 0·0094 | 0·0017 | 1·9E-08 | 0·2113869 | 0·0538427 | 8·64E-05 |
| rs10750025 | 11 | 113424042 | Intergenic | T | C | 0·686 | 0·0103 | 0·0016 | 4·9E-11 | 0·2821195 | 0·0512805 | 3·77E-08 |
| rs11030084 | 11 | 27643725 | Intron:BDNF-AS│LINC00678 | C | T | 0·816 | 0·0106 | 0·0019 | 1·7E-08 | 0·3539683 | 0·0605951 | 5·18E-09 |
| rs12795042 | 11 | 133658168 | Intron:LOC646522 | A | C | 0·377 | 0·0083 | 0·0015 | 3·3E-08 | 0·0456916 | 0·0500773 | 3·62E-01 |
| rs1713676 | 11 | 113660576 | Intergenic | A | G | 0·478 | 0·0080 | 0·0015 | 4·3E-08 | 0·2095090 | 0·0474583 | 1·01E-05 |
| rs4938230 | 11 | 116075001 | Intergenic | A | C | 0·842 | 0·0128 | 0·0020 | 1·5E-10 | 0·2904923 | 0·0666934 | 1·33E-05 |
| rs56030824 | 11 | 47397353 | Intron:SPI1 | G | A | 0·678 | 0·0116 | 0·0016 | 1·2E-13 | 0·3470387 | 0·0505252 | 6·50E-12 |
| rs682011 | 11 | 121544285 | Intergenic | C | T | 0·559 | 0·0082 | 0·0015 | 2·2E-08 | 0·2795524 | 0·0475362 | 4·09E-09 |
| rs7950166 | 11 | 8642218 | Intron:TRIM66 | C | T | 0·363 | 0·0098 | 0·0015 | 9·9E-11 | 0·0754809 | 0·0493386 | 1·26E-01 |
| rs10506274 | 12 | 81601464 | Intron:ACSS3 | G | T | 0·516 | 0·0090 | 0·0015 | 5·8E-10 | 0·2351595 | 0·0473538 | 6·84E-07 |
| rs10876188 | 12 | 51895882 | Intron:SLC4A8 | C | T | 0·543 | 0·0080 | 0·0015 | 4·8E-08 | 0·1807529 | 0·0474407 | 1·39E-04 |
| rs3809162 | 12 | 54674235 | Intergenic | G | A | 0·397 | 0·0091 | 0·0015 | 1·2E-09 | 0·1221380 | 0·0481339 | 1·12E-02 |
| rs4842786 | 12 | 92170791 | Intergenic | G | A | 0·416 | 0·0088 | 0·0015 | 2·7E-09 | 0·1436599 | 0·0482693 | 2·92E-03 |
| rs500321 | 13 | 27124360 | Intergenic | A | T | 0·264 | 0·0097 | 0·0017 | 4·9E-09 | 0·2417876 | 0·0532748 | 5·67E-06 |
| rs1123285 | 14 | 57274519 | Intron:OTX2 | C | G | 0·665 | 0·0089 | 0·0015 | 8·1E-09 | 0·2208075 | 0·0512874 | 1·67E-05 |
| rs11625650 | 14 | 104610138 | Intron:KIF26A | G | A | 0·767 | 0·0096 | 0·0017 | 2·9E-08 | 0·1475926 | 0·0554121 | 7·73E-03 |
| rs2180870 | 14 | 58782779 | Intron:ARID4A | T | C | 0·865 | 0·0122 | 0·0021 | 1·1E-08 | 0·1479222 | 0·0702213 | 3·52E-02 |
| rs28929474 | 14 | 94844947 | Nonsynonymous:SERPINA1 | C | T | 0·982 | 0·0368 | 0·0054 | 1·3E-11 | 0·9382610 | 0·1673706 | 2·07E-08 |
| rs12907323 | 15 | 86796012 | Intron:AGBL1 | G | A | 0·411 | 0·0085 | 0·0015 | 9·9E-09 | 0·1408456 | 0·0490915 | 4·12E-03 |
| rs2472297 | 15 | 75027880 | Intergenic | T | C | 0·249 | 0·0106 | 0·0017 | 3·1E-10 | 0·1312119 | 0·0533459 | 1·39E-02 |
| rs1104608 | 16 | 73912588 | Intergenic | G | C | 0·575 | 0·0110 | 0·0015 | 1·1E-13 | 0·2740378 | 0·0484817 | 1·58E-08 |
| rs113443718 | 16 | 29892184 | Intron:SEZ6L2 | G | A | 0·695 | 0·0102 | 0·0016 | 1·2E-10 | 0·2981284 | 0·0515936 | 7·55E-09 |
| rs17177078 | 16 | 24810681 | Intron:TNRC6A | C | T | 0·937 | 0·0223 | 0·0030 | 1·3E-13 | 0·4741786 | 0·1031741 | 4·31E-06 |
| rs2764771 | 16 | 20013793 | Intergenic | A | G | 0·307 | 0·0099 | 0·0016 | 4·0E-10 | 0·0927403 | 0·0516785 | 7·27E-02 |
| rs378421 | 16 | 28754684 | Intergenic | G | A | 0·596 | 0·0112 | 0·0015 | 4·8E-14 | 0·1831663 | 0·0487283 | 1·71E-04 |
| rs62044525 | 16 | 64872590 | Intergenic | C | G | 0·816 | 0·0122 | 0·0019 | 1·0E-10 | 0·1994346 | 0·0604259 | 9·65E-04 |
| rs7185555 | 16 | 69131281 | Intergenic | G | C | 0·847 | 0·0111 | 0·0020 | 4·2E-08 | 0·1649579 | 0·0649900 | 1·11E-02 |
| rs79616692 | 16 | 72338507 | Intron:LINC01572 | C | G | 0·108 | 0·0163 | 0·0024 | 4·1E-12 | 0·3455765 | 0·0770233 | 7·24E-06 |
| . | 17 | 44246624 | Intron:KANSL1 | C | A | 0·785 | 0·0218 | 0·0026 | 1·6E-17 | 0·5141542 | 0·0566033 | 1·06E-19 |
| rs10438820 | 17 | 78524597 | Intron:RPTOR | T | C | 0·702 | 0·0090 | 0·0016 | 1·8E-08 | 0·1377722 | 0·0514255 | 7·38E-03 |
| rs2854334 | 17 | 29715500 | Intergenic | G | A | 0·615 | 0·0092 | 0·0015 | 7·5E-10 | 0·1476637 | 0·0489807 | 2·57E-03 |
| rs3803800 | 17 | 7462969 | Nonsynonymous:TNFSF12-TNFSF13│TNFSF13 | G | A | 0·786 | 0·0114 | 0·0018 | 1·5E-10 | 0·2193685 | 0·0579716 | 1·54E-04 |
| rs4548913 | 17 | 2209888 | Intron:SRR | G | A | 0·368 | 0·0084 | 0·0015 | 3·1E-08 | 0·1386186 | 0·0497797 | 5·36E-03 |
| rs4092465 | 18 | 55080437 | Intergenic | A | G | 0·365 | 0·0083 | 0·0015 | 4·4E-08 | 0·1805309 | 0·0499899 | 3·05E-04 |
| rs9950000 | 18 | 53052169 | Intron:TCF4 | C | T | 0·605 | 0·0091 | 0·0015 | 9·4E-10 | 0·1542638 | 0·0491566 | 1·70E-03 |
| rs281379 | 19 | 49214274 | Intergenic | A | G | 0·508 | 0·0137 | 0·0015 | 4·9E-21 | 0·2417573 | 0·0475030 | 3·60E-07 |
| rs4815364 | 20 | 25035711 | Intron:ACSS1 | A | G | 0·616 | 0·0086 | 0·0015 | 1·0E-08 | 0·2210707 | 0·0488108 | 5·92E-06 |
| rs9607814 | 22 | 41946519 | Intergenic | C | A | 0·8 | 0·0102 | 0·0019 | 4·3E-08 | 0·3113675 | 0·0600661 | 2·18E-07 |

A1: alcohol consumption(grams/day)-increasing allele; A2: alternative allele; A1F: allele frequency for A1; SE: standard error; SNP: single nucleotide polymorphism; CHR: chromosome number; BP: base-pair position, Genome Reference Consortium Human Build 37 (GRCh37); ^1^Genetic instrument obtained from Liu, et al 2019 of GSCAN GWAS meta-analysis^3^, and we used to construct the alcohol genetic score. ^2^SNPs of alcohol consumption increasing allele in the UK Biobank. Note that alcohol consumption was measured in grams per day.  

## Supplementary Table S3: All-Cause and Cause-specific-mortality by baseline characteristics in the UK Biobank.

|  | **(%)** | **All-Cause Mortality** | **All-Cause Mortality before COVID-19** | **Cancer Mortality** | **CVD Mortality** | **Respiratory Mortality** | **Digestive Mortality** | **COVID-19 Mortality** |
| --- | --- | --- | --- | --- | --- | --- | --- | --- |
|  |  | **n (%)** | **n (%)** | **n (%)** | **n (%)** | **n (%)** | **n (%)** | **n (%)** |
| **Age (years)** |  |  |  |  |  |  |  |  |
| <65 | 222626 (80·05) | 12088 (5·43) | 9184 (4·13) | 6504 (3·00) | 2357 (1·11) | 547 (0·26) | 528 (0·25) | 332(0·16) |
| ≥65 | 55467 (19·95) | 8746 (15·77) | 6445 (11·62) | 4103 (8·07) | 1913 (3·93) | 501 (1·06) | 283 (0·60) | 327(0·67) |
| P^a^ |  | <1·0E-300 | <1·0E-300 | <1·0E-300 | <1·0E-300 | 5·27E-112 | 1·41E-31 | 3·189E-74 |
| **Sex** |  |  |  |  |  |  |  |  |
| Male | 135319 (48·66) | 13154 (9·72) | 9983 (7·38) | 6168 (4·81) | 3107 (2·48) | 678 (0·55) | 552 (0·45) | 444(0·35) |
| Female | 142774 (51·34) | 7680 (5·38) | 5646 (3·95) | 4439 (3·18) | 1163 (0·85) | 370 (0·27) | 259 (0·19) | 215(0·16) |
| P^a^ |  | <1·0E-300 | 5·74E-269 | 5·47E-80 | 5·34E-194 | 1·42E-23 | 1·14E-27 | 1·393E-19 |
| **BMI (kg/m2)** |  |  |  |  |  |  |  |  |
| <18·5 | 1341 (0·48) | 180 (13·42) | 144 (10·74) | 58 (4·76) | 25 (2·11) | 52 (4·29) | 15 (1·28) | 5(0·42) |
| (18·5–25) | 91,655 (33·06) | 5533 (6·04) | 4249 (4·64) | 2960 (3·32) | 907 (1·04) | 361 (0·42) | 208 (0·24) | 108(0·12) |
| (25–30) | 120519 (43·47) | 8709 (7·23) | 6521 (5·41) | 4638 (3·98) | 1758 (1·55) | 350 (0·31) | 282 (0·25) | 274(0·24) |
| ≥30 | 63722 (22·98) | 6213 (9·75) | 4561 (7·16) | 2899 (4·80) | 1532 (2·59) | 270 (0·47) | 298 (0·52) | 264(0·45) |
| P^a^ |  | 1·44E-140 | 3·88E-94 | 1·57E-27 | 7·85E-85 | 1·36E-88 | 3·86E-29 | 1·268E-23 |
| **Smoking** |  |  |  |  |  |  |  |  |
| Non-smokers | 148824 (53·7) | 7746 (5·20) | 5671 (3·81) | 4109 (2·83) | 1491 (1·05) | 211 (0·15) | 264 (0·19) | 237(0·17) |
| Ex-smokers | 100892 (36·4) | 9071 (8·99) | 6844 (6·78) | 4587 (4·76) | 1879 (2·01) | 461 (0·50) | 308 (0·33) | 318(0·34) |
| Current smokers^b^ | 7390 (2·67) | 658 (8·90) | 512 (6·93) | 310 (4·40) | 156 (2·26) | 38 (0·56) | 35 (0·52) | 17(0·25) |
| Cigars/pipes | 1670 (0·6) | 290 (17·37) | 235 (14·07) | 134 (8·85) | 79 (5·41) | 20 (1·43) | 13 (0·93) | 11(0·77) |
| ≤1 to 15 cigs/day | 10966 (3·96) | 1461 (13·32) | 1108 (10·10) | 716 (7·01) | 308 (3·14) | 148 (1·53) | 79 (0·82) | 41(0·42) |
| >15 cigs/day | 7423 (2·68) | 1473 (19·84) | 1162 (15·65) | 698 (10·50) | 322 (5·13) | 156 (2·55) | 108 (1·78) | 29(0·46) |
| P^a^ |  | <1·0E-300 | <1·0E-300 | <1·0E-300 | 4·20E-216 | 1·07E-228 | 6·15E-103 | 4·033E-14 |
| **Physical activity** |  |  |  |  |  |  |  |  |
| Light | 81647 (29·99) | 7032 (8·61) | 5305 (6·50) | 3450 (4·42) | 1493 (1·96) | 417 (0·56) | 305 (0·41) | 243(0·32) |
| Moderate | 136225 (50·04) | 9085 (6·67) | 6793 (4·99) | 4817 (3·65) | 1797 (1·39) | 379 (0·30) | 315 (0·25) | 264(0·20) |
| High | 54336 (19·96) | 3760 (6·92) | 2769 (5·10) | 1980 (3·77) | 760 (1·48) | 148 (0·29) | 133 (0·26) | 124(0·24) |
| P^a^ |  | 8·36E-79 | 3·35E-64 | 2·65E-22 | 1·35E-30 | 5·03E-24 | 2·55E-11 | 4·390E-07 |
| **Education** |  |  |  |  |  |  |  |  |
| None | 45694 (16·56) | 6138 (13·43) | 4518 (9·89) | 2805 (6·62) | 1383 (3·38) | 451 (1·13) | 249 (0·63) | 245(0·60) |
| NVQ/CSE/A-levels | 98291 (35·62) | 6663 (6·78) | 5057 (5·14) | 3443 (3·62) | 1346 (1·45) | 315 (0·34) | 285 (0·31) | 172(0·18) |
| Degree/professional | 131960 (47·82) | 7769 (5·89) | 5853 (4·44) | 4247 (3·31) | 1485 (1·18) | 265 (0·21) | 266 (0·21) | 227(0·18) |
| P^a^ |  | 5·41E-161 | 4·20E-110 | 1·05E-38 | 3·54E-62 | 2·44E-56 | 3·03E-17 | 7·723E-15 |
| **Townsend index** |  |  |  |  |  |  |  |  |
| Quartile 1(lowest) | 70647 (25·43) | 4436 (6·28) | 3326 (4·71) | 2420 (3·53) | 852 (1·27) | 154 (0·23) | 141 (0·21) | 119(0·18) |
| Quartile 2 | 70429 (25·36) | 4781 (6·79) | 3545 (5·03) | 2633 (3·86) | 873 (1·31) | 183 (0·28) | 153 (0·23) | 135(0·20) |
| Quartile 3 | 69692 (25·09) | 5011 (7·190 | 3772 (5·41) | 2579 (3·83) | 1025 (1·56) | 230 (0·35) | 189 (0·29) | 144(0·22) |
| Quartile 4(highest) | 66993 (24·12) | 6590 (9·84) | 4973 (7·42) | 2967 (4·68) | 1516 (2·45) | 481 (0·79) | 327 (0·54) | 261(0·42) |
| P^a^ |  | 2·51E-251 | 6·89E-191 | 1·29E-56 | 3·83E-108 | 8·39E-77 | 1·29E-33 | 1·167E-24 |
| **Self-rated health** |  |  |  |  |  |  |  |  |
| Excellent | 47534 (17·15) | 2051 (4·31) | 10505 (3·17) | 1257 (2·69) | 332 (0·72) | 26 (0·06) | 53 (0·12) | 49(0·11) |
| Good | 163846 (59·1) | 9990 (6·10) | 7389 (4·51) | 5631 (3·53) | 1866 (1·20) | 289 (0·19) | 298 (0·19) | 317(0·20) |
| Fair | 55063 (19·86) | 6154 (11·18) | 4630 (8·41) | 2744 (5·31) | 1441 (2·86) | 423 (0·86) | 303 (0·62) | 199(0·39) |
| Poor | 10770 (3·89) | 2485 (23·07) | 1979 (18·38) | 903 (9·83) | 600 (6·75) | 300 (3·49) | 152 (1·80) | 89(1·01) |
| P^a^ |  | <1·0E-300 | <1·0E-300 | 1·60E-282 | <1·0E-300 | <1·0E-300 | 1·76E-136 | 1·007E-51 |
| **Long-term illness** |  |  |  |  |  |  |  |  |
| No | 186033 (68·36) | 9526 (5·12) | 6993 (3·76) | 5558 (3·05) | 1703 (0·96) | 240 (0·14) | 291 (0·16) | 268(0·15) |
| Yes | 86100 (31·64) | 10764 (12·50) | 8230 (9·56) | 4785 (5·97) | 2455 (3·16) | 782 (1·03) | 494 (0·65) | 373(0·48) |
| P^a^ |  | <1·0E-300 | <1·0E-300 | 4·26E-150 | 4·43E-207 | 2·37E-132 | 3·13E-60 | 5·225E-30 |

^a^P-value for all-cause- and cause-specific mortality, P-values were adjusted for age, sex, and assessment centre. ^b^Smokers without information on the types of tobacco that they smoke.

## Supplementary Table S4: Association of Alcohol GRS with baseline characteristic in the UK Biobank.

|  | **N (%)** | **Alcohol-GRS**  **Mean (SD)** |
| --- | --- | --- |
| **Age (years)** |  |  |
| <65 | 222626 (80.05) | 117.86 (5.50) |
| > 65 P^1^ | 55467 (19.95) | 117.81 (5.53) 0.608 |
| **Sex** |  |  |
| Male | 135319 (48.66) | 117.78 (5.51) |
| Female P^1^ | 142774 (51.34) | 117.91 (5.50) 5.65E-10 |
| **BMI (kg/m2)** |  |  |
| <18·5 | 1341 (0·48) | 118·01 (5·62) |
| (18·5–25) | 91655 (32·96) | 117·77 (5·52) |
| (25-30) | 120519 (43·34) | 117·95 (5·46) |
| ≥30 | 63722 (22·91) | 117·95 (5·47) |
| P^1^ |  | 0.236 |
| **Smoking** |  |  |
| Non-smokers | 148824 (53·52) | 117·74 (5·51) |
| Ex-smokers | 100892 (36·28) | 117·92 (5·50) |
| Smokers* | 7390 (2·66) | 118·17 (5·47) |
| Cigars/pipes | 1670 (0·6) | 118·19 (5·49) |
| ≤1 to 15 cigs/day | 10966 (3·94) | 118·17 (5·53) |
| >15 cigs/day | 7423 (2·67) | 118·27 (5·50) |
| P^1^ |  | <1·0E-300 |
| **Physical activity** |  |  |
| Light | 81647 (29·36) | 117·85 (5·51) |
| Moderate | 136225 (48·99) | 117·84 (5·52) |
| High | 54336 (19·54) | 117·84 (5·47) |
| P^1^ |  | 0·824 |
| **Education** |  |  |
| Degree/professional | 131960 (47·45) | 117·8 (5·52) |
| NVQ/CSE/A-levels | 98291 (35·34) | 117·86 (5·5) |
| None of the above | 45694 (16·43) | 117·95 (5·48) |
| P^1^ |  | 0·138 |
| **Townsend index** |  |  |
| Quartile 1(lowest) | 70647 (25·4) | 117·79 (5·53) |
| Quartile 2 | 70429 (25·33) | 117·78 (5·5) |
| Quartile 3 | 69692 (25·06) | 117·84 (5·53) |
| Quartile 4(highest) P^1^ | 66993 (24·09) | 117·99 (5·46) 0.012 |
| **Self-rated health** |  |  |
| Excellent | 47534 (17.15) | 117.80 (5.51) |
| Good | 163846 (59.10) | 117.83 (5.51) |
| Fair | 55063 (19.86) | 117.92 (5.48) |
| Poor | 10770 (3.89) | 117.91 (5.47) |
| P^1^ |  | 0.043 |
| **Long-term illness** |  |  |
| No | 86100 (31.64) | 117.84 (5.51) |
| Yes | 186033 (68.36) | 117.86 (5.51) |
| P^1^ |  | 0.576 |

NVQ, National Vocational Qualification; CSE, Certificate of Secondary Education; A-levels, Advanced levels; SD, standard deviation; Q, quartiles; cig, cigarette.^ 1^P-value adjusted for age, sex, genotyping array, birth location, and assessment centre, and PC1-40. ^*^Current smokers without information on the types of tobacco that they smoke.

## Supplementary Table S5. Conventional nonlinear observational analysis by different covariate adjustments. 

| **Outcomes** | **P value**^**4**^ |
| --- | --- |
| All-cause mortality^1^ | 0.000 |
| All-cause mortality^2^ | 0.000 |
| All-cause mortality^3^ | 0.000 |
| CVD mortality^1^ | 0.000 |
| CVD mortality^2^ | 0.000 |
| CVD mortality^3^ | 0.000 |
| Cancer mortality^1^ | 0.000 |
| Cancer mortality^2^ | 0.000 |
| Cancer mortality^3^ | 0.000 |
| Respiratory mortality^1^ | 0.000 |
| Respiratory mortality^2^ | 0.000 |
| Respiratory mortality^3^ | 0.000 |
| Digestive mortality^1^ | 0.000 |
| Digestive mortality^2^ | 0.000 |
| Digestive mortality^3^ | 0.000 |
| COVID-19 mortality^1^ | 0.773 |
| COVID-19 mortality^2^ | 0.667 |
| COVID-19 mortality^3^ | 0.816 |

^1^Adjusted for age, sex, BMI, smoking, physical activity, education, TDI, perceived health status, long-term illness, assessment centre. ^2^As model 1 but excluding adjustment for BMI. ^3 ^As model 1 but further adjusted for birth location and top 40 PCs. ^4^Fractional polynomial model P-value for nonlinearity test.  

## Supplementary Table S6. The association between alcohol intake and all-cause mortality stratified by age and self-rated health status.  

| **Model with basic adjustment**^**1**^ |  |  |  |  |  |
| --- | --- | --- | --- | --- | --- |
| **Number of drinks** | **All [OR (95% CI)]** | **Age [OR (95% CI)]** |  | **Self-reported health [OR (95% CI)]** |  |
|  |  | **<50 years** | **≥ 50 years** | **Excellent** | **Poor** |
| None | 1.61(1.53,1.70) | 1.84(1.48,2.30) | 1.60(1.52,1.69) | 1.52(1.26,1.83) | 1.23(1.07,1.42) |
| Light | ref. | ref. | ref. | ref. | ref. |
| Moderate^3^ | 0.94(0.90,0.98) | 1.03(0.87,1.22) | 0.93(0.90,0.97) | 1.11(0.98,1.26) | 0.92(0.80,1.06) |
| High | 1.33(1.26,1.40) | 1.89(1.55,2.30) | 1.30(1.23,1.37) | 1.50(1.27,1.76) | 1.43(1.21,1.69) |
| P-trend | 0.0001 | 0.0000 | 0.0045 | 0.0008 | 0.0033 |
| P-quadratic^4^ | 0.000 | 0.001 | 0.000 | 0.08 | 0.000 |
| **Model with further adjustment**^**2**^ |  |  |  |  |  |
| **Number of drinks** | **All [OR (95% CI)]** | **Age [OR (95% CI)]** |  | **Self-reported health [OR (95% CI)]** |  |
|  |  | **<50 years** | **≥ 50 years** | **Excellent** | **Poor** |
| None | 1.27(1.20,1.34) | 1.15(0.90,1.47) | 1.27(1.20,1.35) | 1.41(1.16,1.71) | 1.10(0.94,1.30) |
| Light | ref. | ref. | ref. | ref. | ref. |
| Moderate^3^ | 0.97(0.93,1.02) | 1.02(0.85,1.22) | 0.97(0.93,1.02) | 1.10(0.96,1.24) | 0.93(0.80,1.09) |
| High | 1.21(1.14,1.28) | 1.56(1.26,1.93) | 1.19(1.12,1.26) | 1.36(1.14,1.61) | 1.29(1.06,1.56) |
| P-trend | 0.0008 | 0.0005 | 0.0106 | 0.0173 | 0.057 |
| P-quadratic^4^ | 0.000 | 0.276 | 0.000 | 0.25 | 0.013 |

^1^The logistic model was adjusted with age, sex, Townsend deprivation index (TDI), assessment centre. ^2^The logistic model was further adjusted with age, sex, BMI, BMI^2, smoking, physical activity, education, TDI, self-rated health, long-term illness, assessment centre, and number of medications. ^3^Moderate drinking defined as 1 to <4 units for females and 1 to <5 units for males.^4^P-non-linearity based on a quadratic term for alcohol intake, adjustments as in the logistic model.

## Supplementary Table S7: MR estimates from linear MR analyses further adjusted with smoking and TDI. 

| **Outcome** | **MR Method** | **Beta**^**1**^ | **LCI** | **UCI** | **P-value** |
| --- | --- | --- | --- | --- | --- |
| All-cause mortality | IVW | 0.0214 | 0.0110 | 0.0318 | 0.0001 |
| All-cause mortality | MR-Egger | 0.0277 | 0.0116 | 0.0438 | 0.0009 |
| All-cause mortality | MR-PRESSO | 0.0231 | 0.0131 | 0.0332 | 0.0000 |
| All-cause mortality | W-Median | 0.0277 | 0.0136 | 0.0419 | 0.0001 |
| All-cause mortality | W-Mode | 0.0283 | 0.0152 | 0.0413 | 0.0001 |
| CVD mortality | IVW | 0.0288 | 0.0094 | 0.0482 | 0.0036 |
| CVD mortality | MR-Egger | 0.0376 | 0.0071 | 0.0680 | 0.0162 |
| CVD mortality | MR-PRESSO | 0.0288 | 0.0091 | 0.0485 | 0.0046 |
| CVD mortality | W-Median | 0.0403 | 0.0112 | 0.0695 | 0.0067 |
| CVD mortality | W-Mode | 0.0389 | 0.0113 | 0.0665 | 0.0070 |
| Cancer mortality | IVW | 0.0151 | 0.0032 | 0.0270 | 0.0132 |
| Cancer mortality | MR-Egger | 0.0178 | -0.0007 | 0.0363 | 0.0586 |
| Cancer mortality | MR-PRESSO | 0.0151 | 0.0030 | 0.0272 | 0.0150 |
| Cancer mortality | W-Median | 0.0135 | -0.0068 | 0.0338 | 0.1922 |
| Cancer mortality | W-Mode | 0.0114 | -0.0057 | 0.0285 | 0.1943 |
| Respiratory mortality | IVW | 0.0046 | -0.0344 | 0.0436 | 0.8176 |
| Respiratory mortality | MR-Egger | -0.0088 | -0.0693 | 0.0517 | 0.7744 |
| Respiratory mortality | MR-PRESSO | 0.0046 | -0.0349 | 0.0441 | 0.8182 |
| Respiratory mortality | W-Median | 0.0401 | -0.0175 | 0.0978 | 0.1726 |
| Respiratory mortality | W-Mode | 0.0061 | -0.0471 | 0.0593 | 0.8225 |
| Digestive mortality | IVW | 0.0754 | 0.0235 | 0.1273 | 0.0044 |
| Digestive mortality | MR-Egger | 0.0957 | 0.0101 | 0.1813 | 0.0289 |
| Digestive mortality | MR-PRESSO | 0.0877 | 0.0382 | 0.1372 | 0.0007 |
| Digestive mortality | W-Median | 0.1131 | 0.0408 | 0.1854 | 0.0022 |
| Digestive mortality | W-Mode | 0.1022 | 0.0378 | 0.1666 | 0.0025 |
| COVID-19 mortality | IVW | 0.0258 | -0.0225 | 0.0741 | 0.2958 |
| COVID-19 mortality | MR-Egger | 0.0201 | -0.0552 | 0.0954 | 0.5975 |
| COVID-19 mortality | MR-PRESSO | 0.0258 | -0.0232 | 0.0747 | 0.2985 |
| COVID-19 mortality | W-Median | 0.0019 | -0.0764 | 0.0803 | 0.9611 |
| COVID-19 mortality | W-Mode | -0.0167 | -0.0892 | 0.0559 | 0.6538 |

^1^Linear MR estimates per one gram per day change of genetically predicted alcohol intake. The analyses were adjusted for age, sex, assessment centre, birth location, SNP array, top 40 PCs, smoking and TDI.

## Supplementary Table S8. Nonlinear MR test for nonlinearity across the varying number of strata.

| **Outcome** | **Number of strata** | **Non-linearity p-value** |
| --- | --- | --- |
| All-cause mortality | 25 | 1 |
|  | 3 | 1 |
|  | 5 | 1 |
|  | 10 | 1 |
|  | 50 | 1 |
| CVD mortality | 25 | 1 |
|  | 3 | 1 |
|  | 5 | 1 |
|  | 10 | 1 |
|  | 50 | 1 |
| Cancer mortality | 25 | 0.5 |
|  | 3 | 0.48 |
|  | 5 | 0.32 |
|  | 10 | 0.54 |
|  | 50 | 1 |
| Respiratory mortality | 25 | 1 |
|  | 3 | 0.23 |
|  | 5 | 0.3 |
|  | 10 | 1 |
|  | 50 | 1 |
| Digestive mortality | 25 | 1 |
|  | 3 | 0.4 |
|  | 5 | 1 |
|  | 10 | 1 |
|  | 50 | 0.52 |
| COVID-19 mortality | 25 | 0.21 |
|  | 3 | 0.4 |
|  | 5 | 0.36 |
|  | 10 | 0.32 |
|  | 50 | 0.27 |

 The analyses were adjusted for age, sex, assessment centre, birth location, types of genotyping array, and the   first 40   PCs. 

 

## Supplementary Figure S1. Diagram for Mendelian randomisation  assumption. 

Valid causal inference from the MR analysis relies on 3 key assumptions: (1) Alcohol-GRS associates with Alcohol consumption; 2) Alcohol-GRS has no direct effect on mortality; 3) Alcohol-GRS does not associate with confounders of alcohol consumption and mortality. Alcohol-GRS: Alcohol consumption genetic risk score. g/day – grams per day.

**Conventional observational  and genetic analysis**

(N = 278,093 participants) 

**Eligible sample** 

(N = 337,463 participants)

**Participants exclusion (N= 165,026)**^**2**^ **based on**

- Non-White British (N= 92,888)

- Reported and genetic sex mismatch (N=312)

- Related participants^3^ (N = 71,826)

**Participants exclusion (N= 59,370) based on**

- Missing information on alcohol consumption, mortality, confounders, and genetic data.  

**UK Biobank sample**

(N= 502,489 participants^1^) 

## Supplementary Figure S2: Participants flow chart of study. 

^1^During analysis, participants who withdrew consent after enrolling in the study were excluded. ^2^Exclusion of participants were performed in sequential order of the list. ^3^Patterns of relatedness were derived from genotype data; one member from each family were included in this study. 

## Supplementary Figure S3. Schematic presentation of the analytical approach.

MR - Mendelian randomisation ; GRS - genetic risk score; SNP - single nucleotide polymorphism; S1-S25, the n^th^ stratum; MRE - MR estimates; NLMRE - non-linear MR estimates; GRS-X, beta of GRS-exposure association; GRS-Y, beta of GRS-outcome association; ^b^beta of SNP-exposure association; ^c^beta of SNP-outcome association; M2, based on doubly-ranked method 25 stratum were constructed; ^d^stratification method to create strata, the detail is elsewhere^4^;  Xmn- average of exposure in the n^th^ stratum; M3, the causal estimate meta-regressed against the mean alcohol consumption level of each stratum; IVW - Inverse Variance Weighted MR, W-Median - Weighted median MR, W-Mode- Weighted mode MR, and MR-PRESSO - Mendelian Randomisation  Pleiotropy RESidual Sum and Outlier.

## Supplementary Figure S4. Conventional nonlinear observational association between alcohol intake and mortality 

The figure shows the associations between alcohol consumption and (A) All-cause mortality before COVID-19 pandemic (covering total deaths up to January 1, 2020) (B) cardiovascular disease mortality; (C) cancer mortality; (D) respiratory mortality; and (E) digestive mortality in the UK Biobank. The dots in the figures represent the reference point (8 grams of daily alcohol intake), and the shaded regions are the 95% confidence intervals. The analyses are adjusted for sex, age, assessment centre, birth location, educational status, TDI, BMI, physical activity, and smoking.

## Supplementary Figure S5: The doubly-ranked method for nonlinear MR estimates of all-cause mortality before onset of covid-19 by the average level of alcohol intake.  

All-cause mortality before COVID-19 (covering total deaths up to January 1, 2020). The effect estimate is in odds ratio with the 95% confidence intervals falls in the shaded area, and the dot represent the reference point of alcohol consumption. Associations were adjusted for age, sex, assessment centre, birth location, SNP array, and the top 40 genetic principal components. CVD-cardiovascular disease 

## Supplementary Figure S6. The estimated genetic association with alcohol consumption at each stratum. 

Y-axis stands for the coefficients between grams of alcohol consumed per day regressed against the GRS. 

## Supplementary Figure S7. The distribution of alcohol GRS across the strata. 

## Supplementary Figure S8. The distribution of alcohol consumption (g/day) across the strata.

(A) 

(B)

(C)

(D)

**(E)**

 

**(F)** 

**(G)**

## Supplementary Figure S9: Association between alcohol-GRS and mortality risk across the strata. 

(A) All-cause mortality (total deaths up to Nov 12, 2021); (B) All-cause mortality before COVID-19 (covering total deaths before the COVID-19 pandemic, up to January 1, 2020); (C) CVD mortality; (D) Cancer mortality; (E) Digestive mortality; (F) Respiratory mortality; (G) COVID-19 mortality in the UK Biobank. The dot is the point estimate, and the vertical bar is the 95% confidence interval. CVD-cardiovascular disease   

(A)

(B)

(C)

(D)

(E)

(F)

(G)

## Supplementary Figure S10. Local average causal effect (LACE) estimates across the strata. 

(A) All-cause mortality (total deaths up to Nov 12, 2021); (B) All-cause mortality before COVID-19 (covering total deaths before the COVID-19 pandemic, up to January 1, 2020); (C) CVD mortality; (D) Cancer mortality; (E) Digestive mortality; (F) Respiratory mortality; (G) COVID-19 mortality in the UK Biobank.  CVD-cardiovascular disease    

 

(A)

(B)

(C)

	(D)

(E)

(F)

(G)

## Supplementary Figure S11.  LACE estimates of mortality against the mean level of alcohol intake in each stratum. 

(A) All-cause mortality (total deaths up to Nov 12, 2021); (B) All-cause mortality before COVID-19 (covering total deaths before the COVID-19 pandemic, up to January 1, 2020); (C) CVD mortality; (D) Cancer mortality; (E) Digestive mortality; (F) Respiratory mortality; (G) COVID-19 mortality in the UK Biobank. The y-axis represented the LACE of the mortality risk per stratum including the 95% confidence interval.  Note that the y-axis refers the LACE estimate of all-cause- and cause-specific mortality per stratum. The error bars represent the 95% confidence interval for each stratum-specific estimate. CVD-cardiovascular disease.

## References 

1.	Department of Health. Alcohol Guidelines Review–Report From the Guidelines Development Group to the UK Chief Medical Officers. Department of Health London; 2016.

2.	Bycroft C, Freeman C, Petkova D, et al. The UK Biobank resource with deep phenotyping and genomic data. *Nature* 2018; 562: 203-9.

3.	Liu M, Jiang Y, Wedow R, et al. Association studies of up to 1.2 million individuals yield new insights into the genetic etiology of tobacco and alcohol use. *Nat Genet* 2019; 51: 237-44.

4.	Tian H, Mason AM, Liu C, Burgess S. Relaxing parametric assumptions for non-linear Mendelian randomization using a doubly-ranked stratification method. *PLoS Genet* 2023; 19: e1010823.

5.	Burgess S, Davies NM, Thompson SG. Bias due to participant overlap in two-sample Mendelian randomization. *Genet Epidemiol* 2016; 40: 597-608.

6.	Burgess S, Thompson SG. *Mendelian randomization: methods for causal inference using genetic variants*. Second ed: Chapman & Hall/CRC, Boca Raton; 2021.

7.	Burgess S, Thompson SG. Avoiding bias from weak instruments in Mendelian randomization studies. *Int J Epidemiol* 2011; 40: 755-64.

8.	Haworth S, Mitchell R, Corbin L, et al. Apparent latent structure within the UK Biobank sample has implications for epidemiological analysis. *Nature Communications* 2019; 10: 333.

9.	Townsend P, Beattie A, Phillimore P. *Health and Deprivation: Inequality and the*

*North. London: Routledge; 1987.*

10.	Bowden J, Del Greco MF, Minelli C, Davey Smith G, Sheehan N, Thompson J. A framework for the investigation of pleiotropy in two-sample summary data Mendelian randomization. *Stat Med* 2017; 36: 1783-802.

11.	Bowden J, Davey Smith G, Burgess S. Mendelian randomization with invalid instruments: effect estimation and bias detection through Egger regression. *Int J Epidemiol* 2015; 44: 512-25.

12.	Burgess S, Thompson SG. Interpreting findings from Mendelian randomization using the MR-Egger method. *Eur J Epidemiol* 2017; 32: 377-89.

13.	Bowden J, Davey Smith G, Haycock PC, Burgess S. Consistent Estimation in Mendelian Randomization with Some Invalid Instruments Using a Weighted Median Estimator. *Genet Epidemiol* 2016; 40: 304-14.

14.	Hartwig FP, Davey Smith G, Bowden J. Robust inference in summary data Mendelian randomization via the zero modal pleiotropy assumption. *Int J Epidemiol* 2017; 46: 1985-98.

15.	Verbanck M, Chen CY, Neale B, Do R. Detection of widespread horizontal pleiotropy in causal relationships inferred from Mendelian randomization between complex traits and diseases. *Nat Genet* 2018; 50: 693-8.

16.	Staley JR, Burgess S. Semiparametric methods for estimation of a nonlinear exposure-outcome relationship using instrumental variables with application to Mendelian randomization. *Genet Epidemiol* 2017; 41: 341-52.

17.	Burgess S. Violation of the Constant Genetic Effect Assumption Can Result in Biased Estimates for Non-Linear Mendelian Randomization. *Human Heredity* 2023; 88: 79-90.

18.	Hamilton FW, Hughes DA, Spiller W, Tilling K, Smith GD. Non-linear mendelian randomization: evaluation of biases using negative controls with a focus on BMI and Vitamin D. *medRxiv* 2023: 2023.08.21.23293658.

19.	Burgess S, Sun Y-Q, Zhou A, Buck C, Mason AM, Mai X-M. Body mass index and all-cause mortality in HUNT and UK Biobank studies: revised non-linear Mendelian randomization analyses. *medRxiv* 2023: 2023.10.31.23297612.

20.	Stockwell T, Zhao J, Panwar S, Roemer A, Naimi T, Chikritzhs T. Do "Moderate" Drinkers Have Reduced Mortality Risk? A Systematic Review and Meta-Analysis of Alcohol Consumption and All-Cause Mortality. *J Stud Alcohol Drugs* 2016; 77: 185-98.

21.	Hemani G, Zheng J, Elsworth B, et al. The MR-Base platform supports systematic causal inference across the human phenome. *Elife* 2018; 7.

22.	Mason AM, Burgess S. Software Application Profile: SUMnlmr, an R package that facilitates flexible and reproducible non-linear Mendelian randomization analyses. *International Journal of Epidemiology* 2022; 51: 2014-9.
